# Identification of different proteins binding to Na, K-ATPase α1 in LPS-induced ARDS cell model by proteomic analysis

**DOI:** 10.1186/s12953-022-00193-3

**Published:** 2022-06-09

**Authors:** Xu-Peng Wen, Guo Long, Yue-Zhong Zhang, He Huang, Tao-Hua Liu, Qi-Quan Wan

**Affiliations:** 1 Transplantation Center, the Third Xiangya Hospital, Central South University, Changsha, 410013 Hunan China; 2 Respiratory ICU, the Third Xiangya Hospital, Central South University, Changsha, 410013 Hunan China; 3 Clinical Medicine, Xiangya School of Medicine, Central South University, Changsha, 410083 China; 4 Hunan International Travel Health Care Center, Changsha, 410001 Hunan China

**Keywords:** ARDS, Lipopolysaccharide, Proteomics, Na, K-ATPase α1, A549 cell

## Abstract

**Background:**

Acute respiratory distress syndrome (ARDS) is characterized by refractory hypoxemia caused by accumulation of pulmonary fluid, which is related to inflammatory cell infiltration, impaired tight junction of pulmonary epithelium and impaired Na, K-ATPase function, especially Na, K-ATPase α1 subunit. Up until now, the pathogenic mechanism at the level of protein during lipopolysaccharide- (LPS-) induced ARDS remains unclear.

**Methods:**

Using an unbiased, discovery and quantitative proteomic approach, we discovered the differentially expressed proteins binding to Na, K-ATPase α1 between LPS-A549 cells and Control-A549 cells. These Na, K-ATPase α1 interacting proteins were screened by co-immunoprecipitation (Co-IP) technology. Among them, some of the differentially expressed proteins with significant performance were identified and quantified by liquid chromatography-tandem mass spectrometry (LC–MS/MS). Data are available via ProteomeXchange with identifier PXD032209. The protein interaction network was constructed by the related Gene Ontology (GO) and Kyoto Encyclopedia of Genes and Genomes (KEGG) analysis. Several differentially expressed proteins were validated by Western blot.

**Results:**

Of identified 1598 proteins, 89 were differentially expressed proteins between LPS-A549 cells and Control-A549 cells. Intriguingly, protein–protein interaction network showed that there were 244 significantly enriched co-expression among 60 proteins in the group control-A549. while the group LPS-A549 showed 43 significant enriched interactions among 29 proteins. The related GO and KEGG analysis found evident phenomena of ubiquitination and deubiquitination, as well as the pathways related to autophagy. Among proteins with rich abundance, there were several intriguing ones, including the deubiquitinase (OTUB1), the tight junction protein zonula occludens-1 (ZO-1), the scaffold protein in CUL4B-RING ubiquitin ligase (CRL4B) complexes (CUL4B) and the autophagy-related protein sequestosome-1 (SQSTM1).

**Conclusions:**

In conclusion, our proteomic approach revealed targets related to the occurrence and development of ARDS, being the first study to investigate significant differences in Na, K-ATPase α1 interacting proteins between LPS-induced ARDS cell model and control-A549 cell. These proteins may help the clinical diagnosis and facilitate the personalized treatment of ARDS.

**Graphical Abstract:**

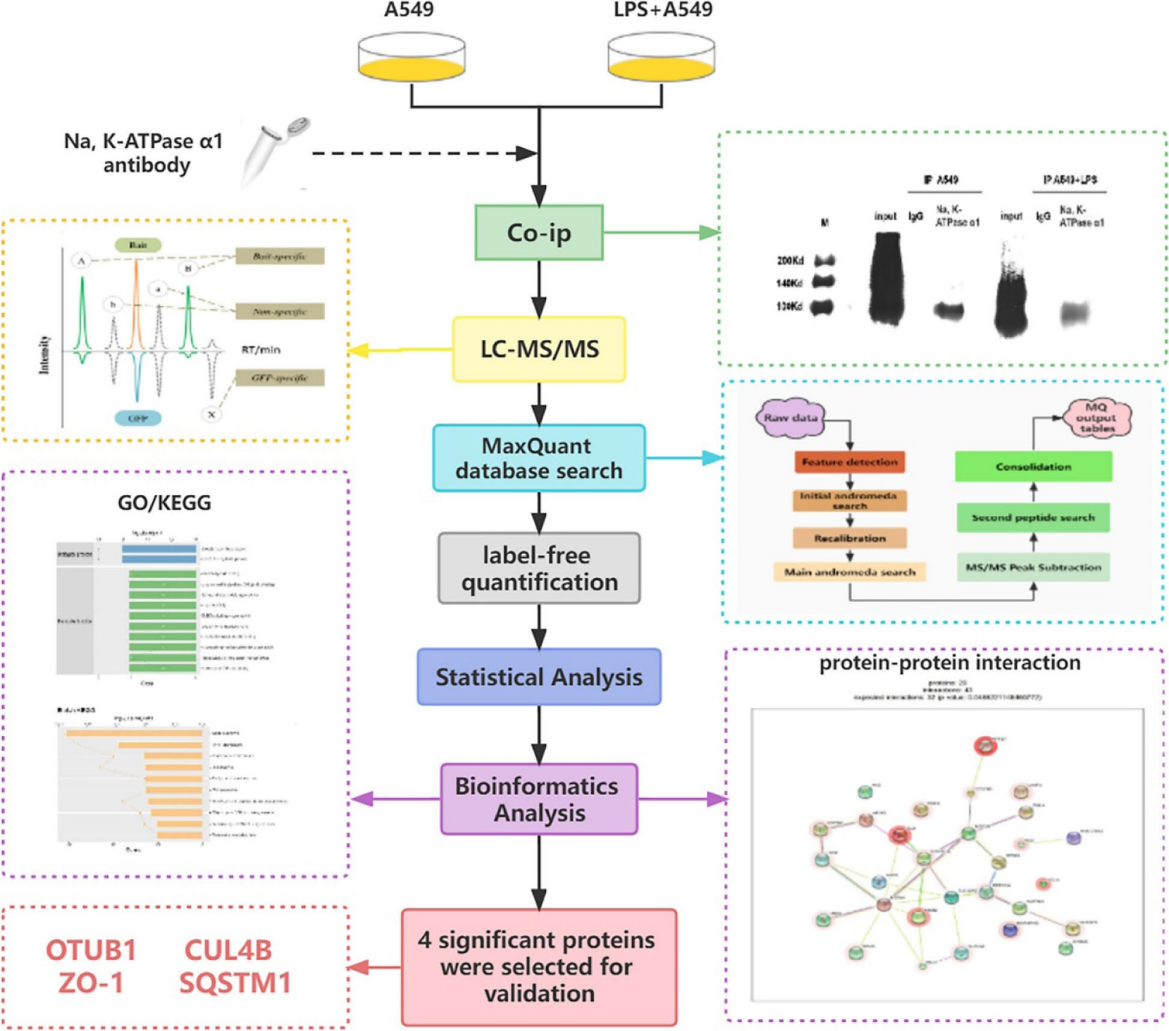

**Supplementary Information:**

The online version contains supplementary material available at 10.1186/s12953-022-00193-3.

## Introduction

Acute respiratory distress syndrome (ARDS) is a potentially fatal clinical syndrome that occurs as a result of diversified pulmonary and extrapulmonary factors, characterized by excessive lung inflammatory response, impaired tight junction of pulmonary epithelium, decreased pulmonary gas exchange ability and reduced alveolar fluid clearance (AFC) of the lungs with consequent refractory hypoxemia [1]. Effective removal of excess edema fluid in the alveoli and maintenance of dry alveolar space are the main ways to relieve ARDS [2]. The apically-located epithelial Na^+^ channel (ENaC) and sodium pump, namely Na, K-ATPase, on the basolateral surface of alveolar type II epithelial cells (AT II) mediated sodium ion transport is the main dynamic of AFC [3]. The imbalance of Na, K-ATPase will aggravate the formation of pulmonary edema by limiting Na^+^ transport and destroying the alveolar barrier function [4].

Na, K-ATPase, is a ubiquitous enzyme consisting of three subunits. Among them, α-subunit plays a key role and is the most important one in sodium-water transport as the main driving force of Na^+^ and K^+^ exchange in the lung to promote fluid clearance in the alveoli. There are four subtypes of α subunit, only α1 exists in lung [5]. Investigating Na, K-ATPase α1-related pathway may provide new strategies and targets for ARDS treatment. But, a powerful tool to precisely and quantitatively detect changes in protein expression in response to ARDS is necessary.

**Fig. 1:**
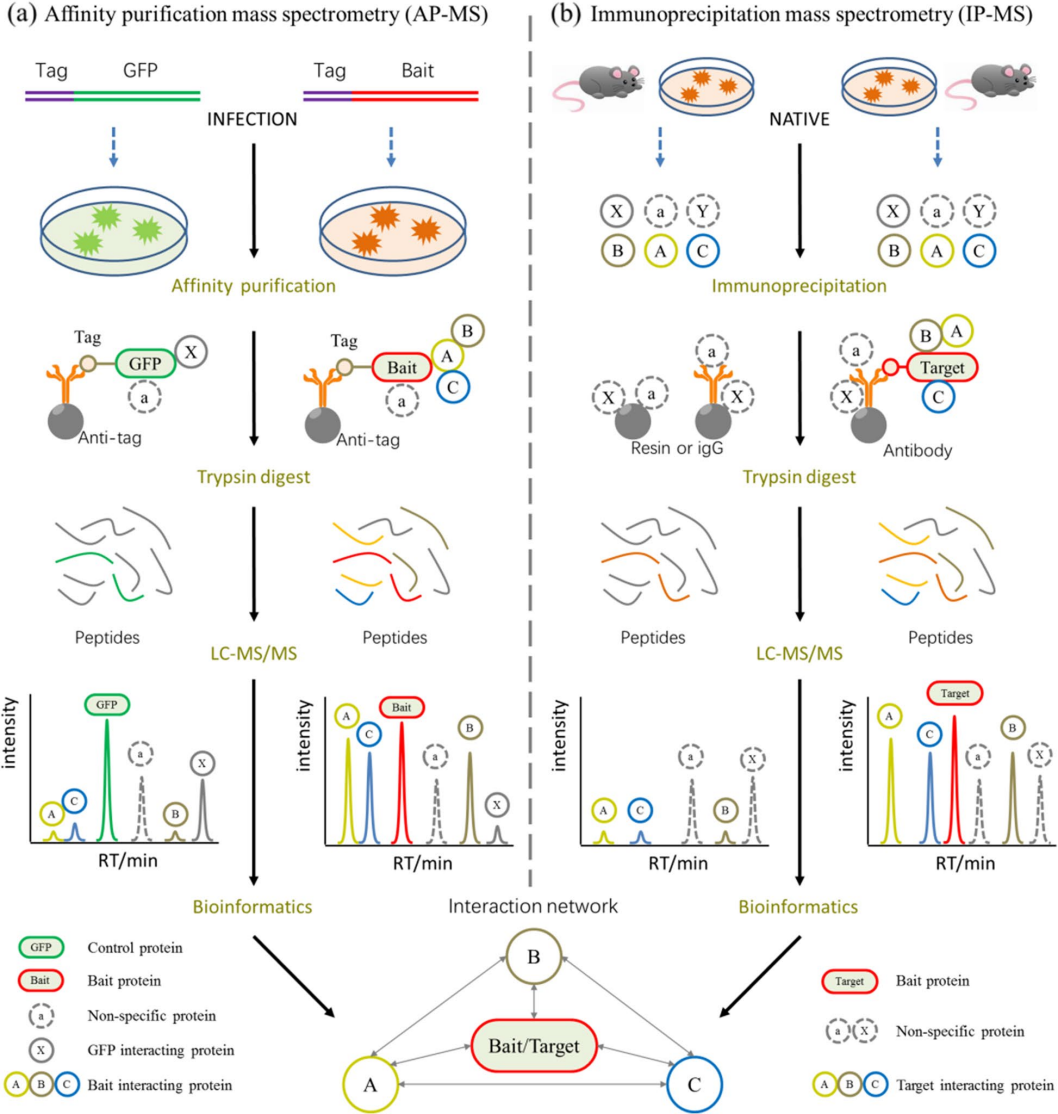
The workflow of AP-MS (IP-MS) technology. Using this approach with no IP-level antibodies are available against the target protein. Target protein(bait) is co-expressed with affinity tag and forms a complex with the endogenous components, then purified with immobilized tag affinity protein dynabeads and identified with LC–MS/MS after extensively washing off unspecifically bound proteins

**Fig. 2:**
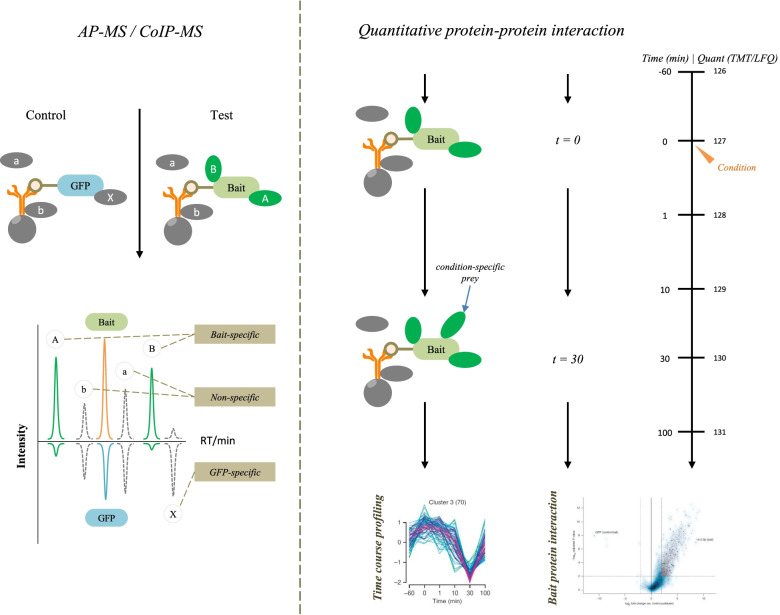
Procedure summary of Co-IP-MS techniques. Using this approach with IP-level antibodies are available against the target protein. For untransfected sample, protein complex is affinity captured from native cell lysates by an immobilized antibody that specifically recognizes an epitope of target (bait) protein. The co-isolated protein complex is washed extensively to remove unspecifically bound proteins and is subsequently eluted from the resin prior to protein identification by mass spectrometry

In the present study, we utilized LPS-induced human AT II cell line (A549) as a model of ARDS [6], and detected the changes in the protein expression profiles of LPS-A549 group compared with control-A549 group and control-IgG group. Currently, the majority of studies on the composition of protein complexes are carried out by affinity purification mass spectrometry (AP-MS/MS), or by co-immunoprecipitation mass spectrometry (Co-IP-MS) for untransfected native samples, and present a static view of the system (Figs. 1 and 2) (free images were obtained from Aksomics). We employed affinity purification (AP) or co-immunoprecipitation (Co-IP) technology to separate endogenous or labeled bait proteins and the proteins interacting with them. Then, we used liquid chromatography-tandem mass spectrometry (LC–MS/MS) technology to identify and quantify these proteins, combined with Gene Ontology (GO) and Kyoto Encyclopedia of Genes and Genomes (KEGG) analysis, constructing the protein interaction network. Altogether, differential protein expression data may provide a valuable resource to reveal potential molecular targets for ARDS treatment.

## Materials and methods

### Reagents

LPS (Escherichia coli serotype 055: B5) was obtained from Sigma-Aldrich (St Louis, MO, USA); Na, K-ATPase α1 antibody and SQSTM1 rabbit polyclonal antibody were purchased from Proteintech (Chicago, USA); CUL4B rabbit polyclonal antibody was purchased from Immunoway (Newark, DE, USA).

### Cell line and cell culture

A549 cell line was purchased from ATCC; A549 cells were seeded into plastic culture dishes at 1 × 10^6/cm2 and cultured in a humidified incubator (21% O2, 5% CO2, 37 °C) in DMEM with 10% fetal bovine serum (FBS), 2 mM L-glutamine, 100 units/ml penicillin, and 100 μg/ml streptomycin. For all experiments, cells were grown and maintained in six-well plates, and cells were serum deprived for 24 h prior to pretreatment with LPS at a concentration of 1 ug/ml for 12 h at 37 °C. The final number of cells we used was 2 × 10^7 A549 cells. This was resuscitated starting from a cryogenic vial, when growing a full T25 culture flask, which was about 1 × 10^6 cells at that point. Whereas 2 × 10^7 A549 cells would take 10 flasks, roughly 2–3 weeks.

### Sample preparation

There were two groups: A: A549 cells, B: A549 cells + LPS (1 μg/ml, cultured for 12 h). ARDS was induced by LPS according to previous reports [7]. Na, K-ATPase α1 antibody was used to pull down the Na, K-ATPase α1 proteins in two groups of cells. These proteins were pulled down for label-free mass spectrometry to understand the binding and interacting protein, and then the protein of interest was selected for verification.

### Co-immunoprecipitation (Co-IP) assay

Fifty microliter anti-igG Dynabeads were used for each sample. Beads were washed 3 times with 500μL PBSN and shaken gently for 1 min. 2 μg antibody or normal IgG were mixed with 200Î¼L PBSN. The cleaned Dynabeads were re-suspended and shaken for 1 h at 4 °C slowly. Free antibody was washed out 3 times with 500μL PBSN and shaken gently for 1 min. Dynabeads were mixed with sample lysate and shaken for 2 h at 4 °C slowly. Then the supernatant was transferred to a new EP tube and stored at -80 °C. Unbinding proteins were washed out with 500μL PBSN and shaken for 1 min. NP-40, which is incompatible with LC–MS, were washed out with 500μL PBS4 times. 50μL 1% TFA was added to Dynabeads and incubated 10 min at 37 °C with highspeed shaking to elute binding proteins. The supernatant was transferred to a new load-binding EP tube. Finally, the elution step was repeated once, combined two elutions, and adjusted to neutral pH with 10% ammonium hydroxide. 100μL ABC buffer was added for trypsin digest.

#### LC–MS/MS

LC–MS/MS -based assays were performed as previously described with some minor alterations [8, 9]. For each sample, ~ 1/2 peptide were separated and analyzed with a nano-UPLC (EASY-nLC1200) coupled to Q-Exactive mass spectrometry (Thermo Finnigan). Separation was performed using a reversed-phase column (100 μm, ID × 15 cm, Reprosil-Pur 120 C18-AQ, 1.9 μm, Dr. Math). Mobile phases were H2O with 0.1% FA, 2% ACN (phase A) and 80% ACN, 0.1% FA (phase B). Separation of sample was executed with a 120 min gradient at 300 nL/min flow rate. Gradient B: 8 to 30% for 92 min, 30 to 40% for 20 min, 40 to 100% for 2 min, 100% for 2 min, 100 to 2% for 2 min and 2% for 2 min. Data dependent acquisition was performed in profile and positive mode with Orbitrap analyzer at a resolution of 70,000 (@200 m/z) and m/z range of 350–1600 for MS1; For MS2, the resolution was set to 17,500 with a dynamic first mass. The automatic gain control (AGC) target for MS1 was set to 1.0 E + 6 with max IT 100 ms, and 5.0 E + 4 for MS2 with max IT 200 ms. The top 10 most intense ions were fragmented by HCD with normalized collision energy (NCE) of 27%, and isolation window of 2 m/z. The dynamic exclusion time window was 20 s. The mass spectrometry proteomics data have been deposited to the ProteomeXchange Consortium via the PRIDE [1] partner repository with the dataset identifier PXD032209.

### MaxQuant database search

The MaxQuant computational platform was performed as described previously [10]. Raw MS files were processed with MaxQuant (Version 1.5.6.0). The protein sequence database (Uniprot_organism_2016_09) was downloaded from UNIPROT.

### Quantification and class specific grouping

We performed proteomic profiling from LPS-A549 cell group and control A549 cell group (Table S1) using the Co-IP and LC–MS/MS technology. In this study, the FDR of polypeptide and protein levels were all controlled at 0.01.

### Bioinformatics analysis

Enrichment of gene ontology (GO) terms was measured [11]. Further, pathway analysis for the differentially expressed proteins was carried out by Kyoto Encyclopedia of Genes and Genomes (KEGG) tool which was performed by STRING analysis (https://string-db.org/) [12]. The interactions of the proteins were also determined by STRING.

### Statistical analysis

All results were expressed as themean ± standard deviation. The normalized spectral count of protein in purification was performed as previously described [13]. And the difference multiple selection, identification and quantitative results were as follows: FCA value > 1 or FCA value < -1, and the protein with unique peptide number ≥ 2 was defined as significant difference. Statistical significance was set at a *P* < 0.05 (*), *P* < 0.01 (**) and *P* < 0.001 (***).

## Results

### Na, K-ATPase α1 antibody successfully pulled down the binding proteins of Na, K-ATPase α1 by Co-IP

**Fig. 3:**
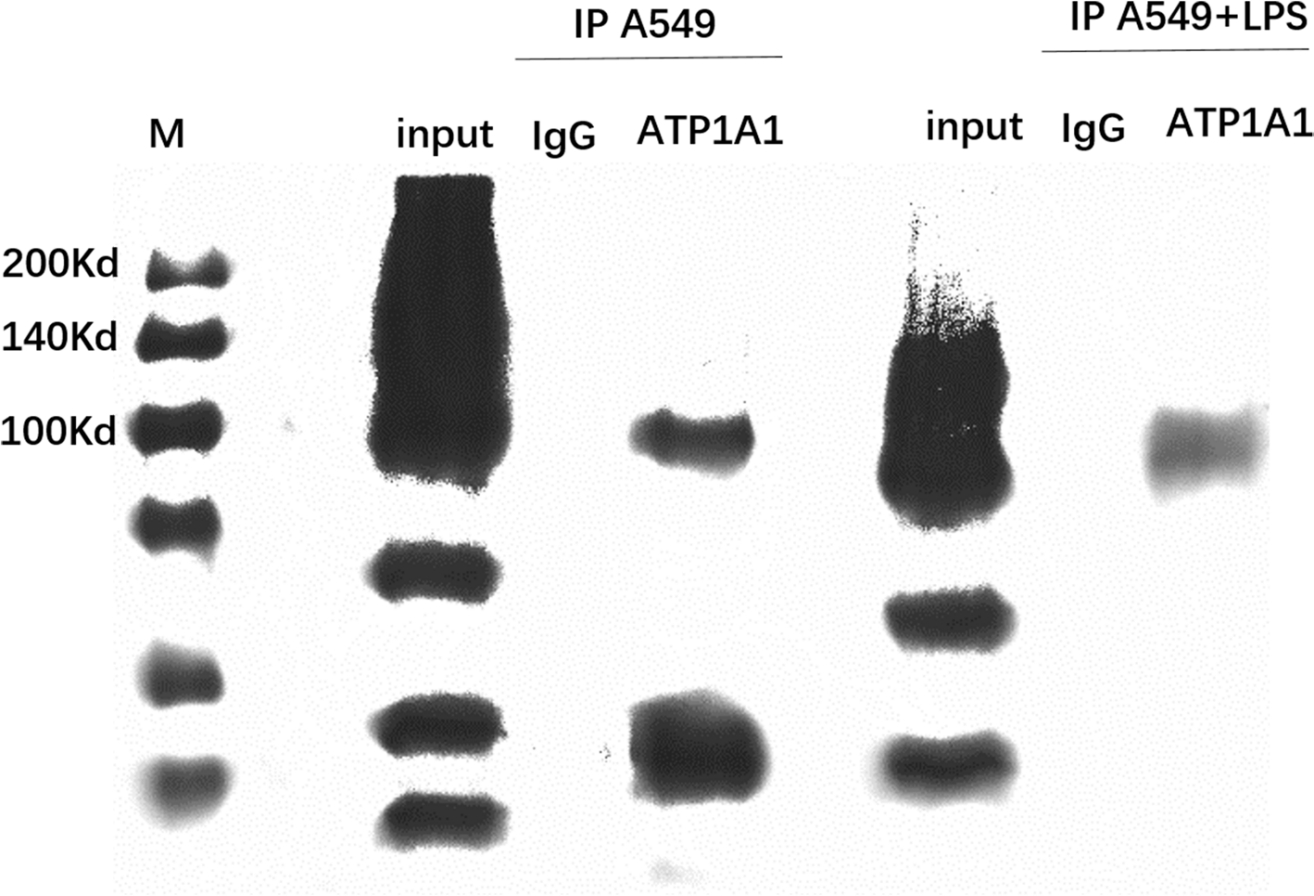
The binding proteins of Na, K-ATPase α1 in A549 group and A549 + LPS group. The binding proteins of Na, K-ATPase α1 was detected in both groups using Co-IP assay followed by Western blot (*P* < 0.001). Proteins in whole-cell lysate were used as a positive control (input). ATP1A1: the gene name of Na, K-ATPase α1

To verify whether there are proteins that can bind to Na, K-ATPase α1, the total proteins were isolated from cell lysates and then detected by Western blot. A549 cells were divided into two groups: the control-A549 and LPS-A549 groups. The protein expression of Na, K-ATPase α1 was detected in both groups (both *p* < 0.001) (Fig. 3). Thus, we concluded that there were proteins that could interact with and bind to Na, K-ATPase α1 in both groups, from which we could start related proteomic analysis and identify exactly related proteins of Na, K-ATPase α1 in the future work.

### Identification and quantification of different proteins

To identify and quantify the significant proteins, in this study, we performed that the total number of identified and quantitated proteins in this assay was 1598, and among them, there are 738 proteins after filtration and 89 differentially expressed proteins in the group LPS-A549 compared with control-A549 (Table S2). Six hundred ninety-eight significant proteins in the group Control-A549 compared with IgG-A549 and 478 significant proteins in the group LPS-A549 compared with IgG-LPS (Table S3).

**Fig. 4:**
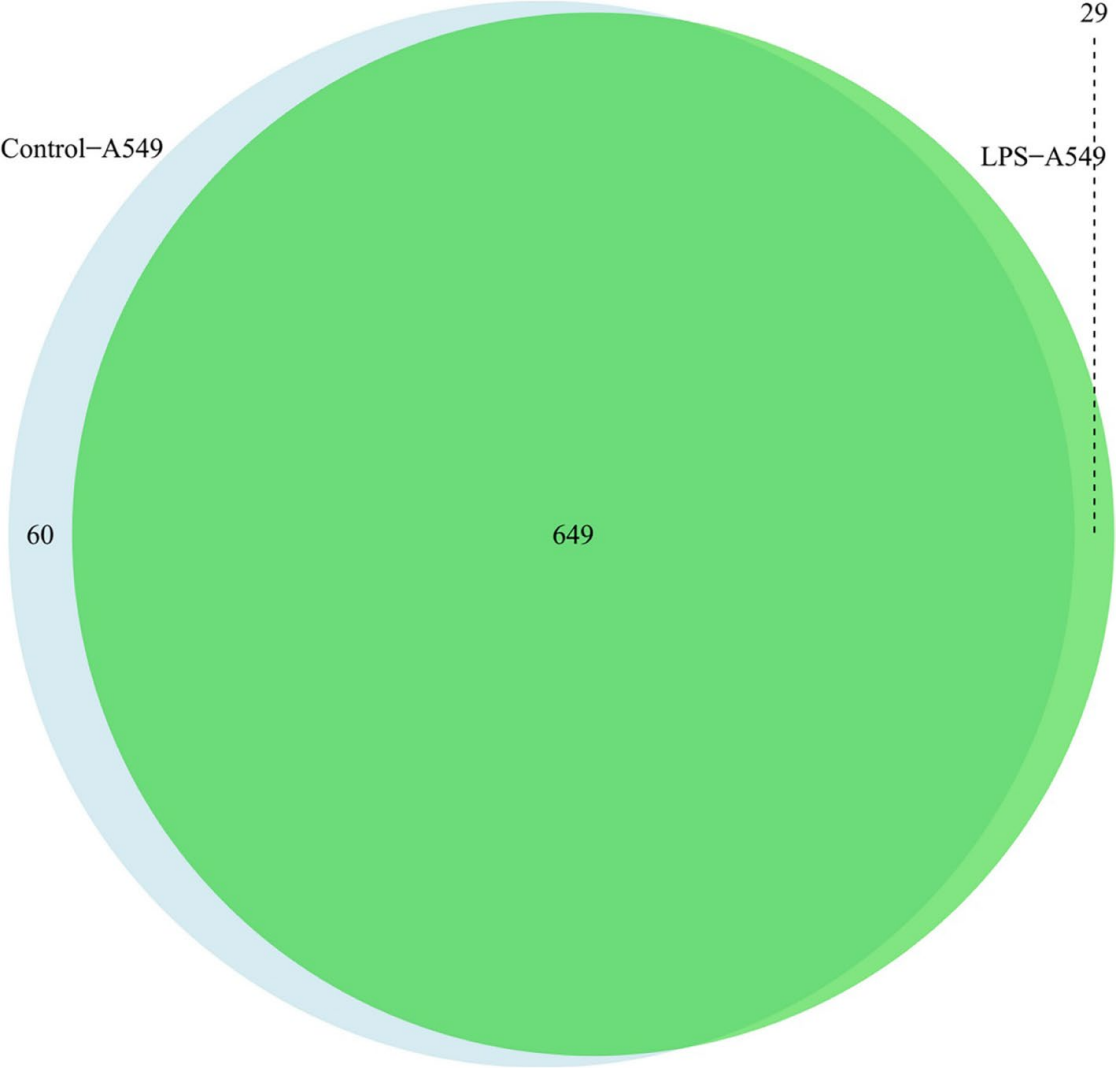
Venn diagram of the different proteins in LPS-A549 vs. control-A549. The green part represents proteins enriched in LPS-A549; The light blue represents proteins enriched in A549; The middle part is the protein identified by both of them

Respectively, Venn diagrams were then drawn to confirm the different proteins in the three comparisons, namely, LPS-A549 vs. control-A549 (Fig. 4), LPS-A549 vs. IgG-LPS (Fig S1) and control-A549 vs. IgG-A549 (Fig S2). In the discovery phase, we identified that 29 proteins were enriched in the LPS-A549 group, 60 proteins were enriched in the Control-A549 group, and 649 proteins were co-enriched in both groups (Fig. 4). We ranked the proteins according to fold change in expression levels and listed the top 10 candidates of significantly up-or down-regulated proteins (Table 1). Also, we found that 738 proteins contained E3 ubiquitin ligase or its complex components: TRIP12, RNF21 and CUL4B, deubiquitinases UCHL1, EIF3F and OTUB1, tight junction protein TJP1, and multifunctional protein SQSTM1. Among them, UCHL1 and RNF213 only enriched in the control group, while CUL4B, TRIP12, EIF3F, TJP1, SQSTM1 and OTUB1 enriched in both groups, indicating that they are strongly bound to Na, K-ATPase α1. These analysis results suggested that the difference in proteomic profiling is reliable. In future work, we can select the proteins we are interested in to carry out some relevant verification.

**Table 1:** Top 10 up-or down-regulated proteins ranking by FC in LPS-A549 group vs. control-A549 group

Rank	LPS-A549 vs Control-A549		
	Annotation	Alias	FC^a^
**Up-regulated Proteins**			
1	keratin 17	KRT17	3.20673671
2	dihydrolipoamide dehydrogenase	DLD	2.619500792
3	nicalin	NCLN	2.355100614
4	mutS homolog 2	MSH2	2.355100614
5	heterogeneous nuclear ribonucleoprotein H3 (2H9)	HNRNPH3	1.619500792
6	stearoyl-CoA desaturase (delta-9-desaturase)	SCD	1.619500792
7	LIM and SH3 protein 1	LASP1	1.451281189
8	coatomer protein complex, subunit beta 2 (beta prime)	COPB2	1.451281189
9	coiled-coil domain containing 86	CCDC86	1.355100614
10	solute carrier family 1 (neutral amino acid transporter), member 5	SLC1A5	1.355100614
**Down-regulated Proteins**			
1	ribosomal RNA processing 1 homolog B	RRP1B	-3.459431619
2	dolichyl-phosphate mannosyltransferase polypeptide 1	DPM1	-2.584962501
3	GDP dissociation inhibitor 2	GDI2	-2.321928095
4	nucleoporin 107 kDa	NUP107	-2.321928095
5	talin 1	TLN1	-2.321928095
6	WD repeat domain 36	WDR36	-2.321928095
7	WD repeat domain 46	WDR46	-2.149102965
8	DEAH (Asp-Glu-Ala-Asp/His) box polypeptide 57	DHX57	-2.000000000
9	G1 to S phase transition 2	GSPT2	-2.000000000
10	nuclear factor of kappa light polypeptide gene enhancer in B-cells 2	NFKB2	-2.000000000

^a^*FC* Fold Change

### GO & KEGG enrichment analysis of proteins interacting with Na, K-ATPase α1

To search for shared functions among genes, a common way is to incorporate the biological knowledge, by GO and KEGG analysis, to identify predominant biological themes of a collection of genes.

#### GO enrichment

GO analysis (http://www.geneontology.org/) was applied to search for significantly enriched GO terms in areas of biological process (BP), cellular component (CC), and molecular function (MF). Prediction terms with *P*-value less than 0.05 were selected and ranked by gene count ((Count/Pop. Hits)/(List. Total/Pop. Total)) or enrichment score (log10(adjust *p*-value)).

**Fig. 5:**
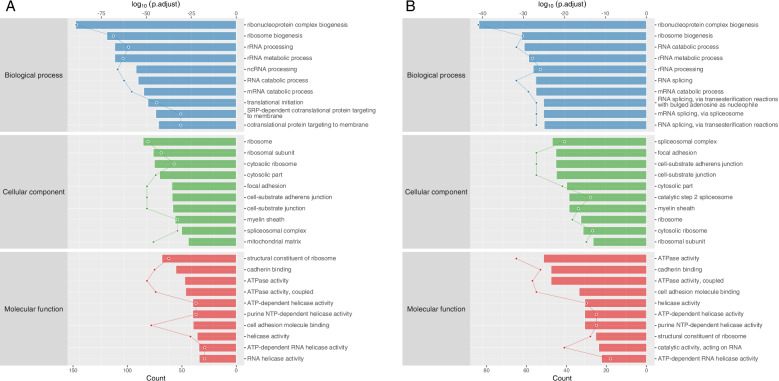
**A** Enriched GO items of < T > in Control-A549 vs. IgG-A549. Top axis is log10 (adjust *p*-value), bottom axis is gene count. **B** Enriched GO items of < T > in LPS-A549 vs. IgG-LPS

According to the results of group Control-A549 vs. IgG-A549, 750 BP terms, 229 CC terms, and 204 MF terms were found enriched in class-specific test enriched (T) sample compared with control enriched (C) sample. Similarly, in line with the results of group LPS-A549 vs. IgG-LPS, 697 BP terms, 202 CC terms, and 177 MF terms were found enriched in T sample. These generally changed GO terms in T sample and classified by BP, CC, MF, and ranked by gene count and enrichment score (Fig. 5A and B) (*p* < 1.0 × 10^−7^ in all terms).

**Fig. 6:**
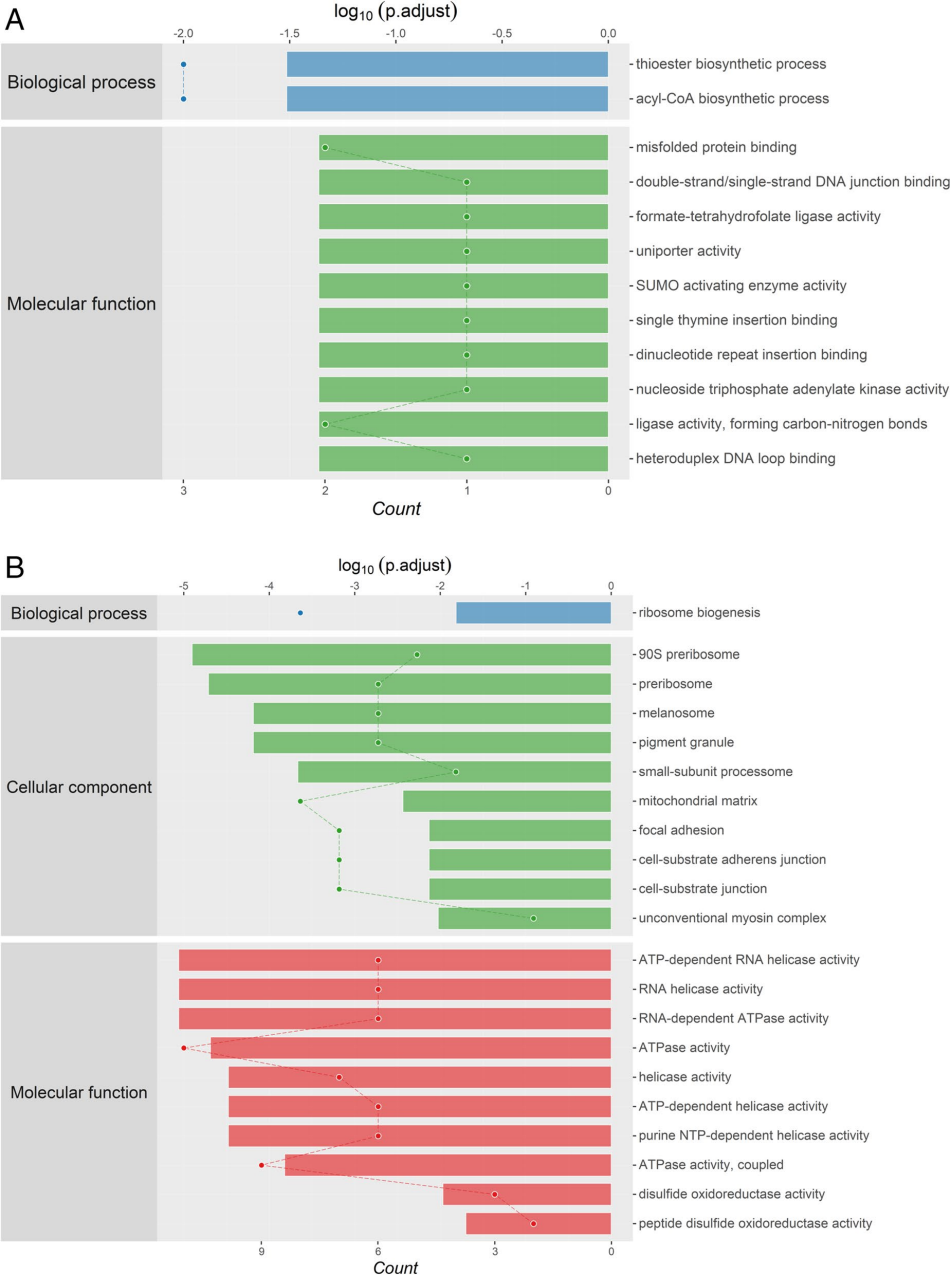
**A** Enriched GO items of < T > in LPS-A549 vs. Control-A549. **B** Enriched GO items of < C > in LPS-A549 vs. Control-A549

Nevertheless, for what we are concerned about the results of group LPS-A549 vs. Control-A549, only 2 BP terms, 0 CC terms, and 40 MF terms were found enriched in T sample compared with C sample (namely, 1 BP terms, 20 CC terms, and 15 MF terms were found enriched) (Fig. 6A and B). Intriguingly, almost all of the most enriched and meaningful BP terms were related to biosynthetic process in the LPS-A549 group, for instance, “thioester biosynthetic process (GO:0,035,384),” “acyl-CoA biosynthetic process (GO:0,071,616),” and only “ribosome biogenesis (GO:0,042,254)” in the Control-A549 group. Some more detailed data can be found in (Fig S3 and S4).

The most enriched CC terms were primarily about the cell in only group control-A549 such as “90S pre-ribosome (GO:0,030,686),” “pre-ribosome (GO:0,030,684),” “melanosome (GO:0,042,470),” “pigment granule (GO:0,048,770),” and “small-subunit processsome (GO:0,032,040).”

As for GO MF terms ranked by either gene count or enrichment score, the mainly enriched terms were closely related to enzymatic activity and protein binding. Represented terms were “misfolded protein binding (GO:0,051,787),” “double-strand/single-strand DNA junction binding (GO:0,000,406),” “formate-tetrahydrofolate ligase activity (GO:0,004,329),” “SUMO activating enzyme activity (GO:0,019,948),” in the LPS-A549 group, and “ATP-dependent RNA helicase activity (GO:0,004,004),” and “ATPase activity (GO:0,016,887).” “ATPase activity, coupled (GO:0,042,623),” in the Control-A549 group.

#### KEGG pathway

We selected differentially expressed proteins for KEGG enrichment analysis, and the results demonstrated that the KEGG pathway was significantly enriched (p.adjust < 0.05). Pathways (p.adjust < 0.05) were selected and ranked by gene counts. Overall, in the group Control-A549 vs. IgG-A549, 689 differentially expressed proteins were involved in 23 KEGG pathways, like Ribosome, Spliceosome and Carbon metabolism. And in the group LPS-A549 vs. IgG-LPS, 478 differentially expressed proteins were involved in 29 KEGG pathways. The proteins were primarily enriched in RNA transport and Fatty acid metabolism (Fig S5 and S6). And top 20 pathways were listed for up-regulated genes, respectively (Table S4).

### STRINGdb protein–protein interaction (PPI) analysis

**Fig. 7:**
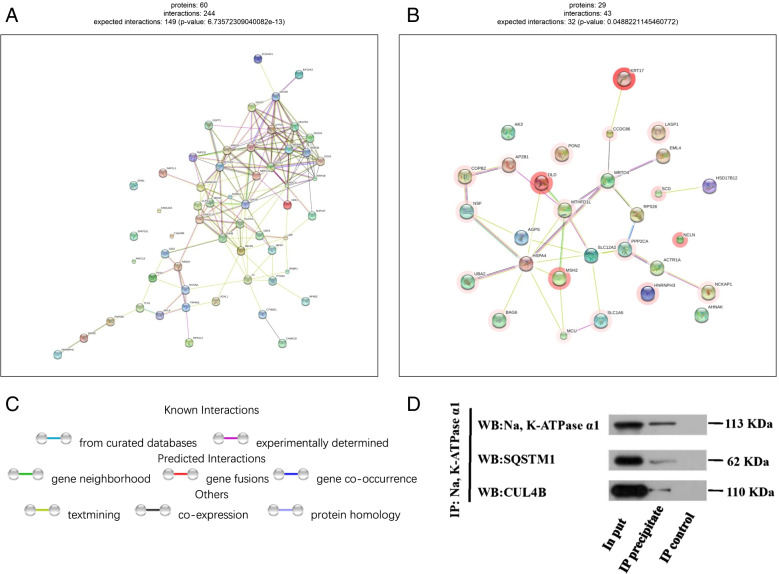
STRING protein–protein interaction analysis. **A** Protein interaction analysis of control-A549 specifically enriched proteins by STRINGdb showed that there were 244 significant enriched interactions among 60 proteins. (p-value: 6.73572309040082e-13). **B** Protein interaction analysis of LPS-A549 specifically enriched proteins by STRINGdb showed that there were 43 significant enriched interactions among 29 proteins. (p-value: 0.0488221145460772). **C** Edge color legends. The explanation of protein–protein interaction links. It is divided into two parts: known interactions and predicted interactions. **D** The binding of Na, K-ATPase α1 to SQSTM1 and CUL4B were verified by endogenous Co-IP. Proteins in whole-cell lysate were used as a positive control (Input). IP: Na, K-ATPase murine monoclonal antibody, abcam, ab2872. Na, K-ATPase α1 group, WB: 1:1000, 100KD. Second antibody: Goat anti-Mouse IgG (Light Chain Specific), HRP Conjugated, S003, 1:5000. SQSTM1 group, WB: SQSTM1 rabbit polyclonal antibody, Proteintech, 18,420–1, 1:10,000, 62KD; Second antibody: Mouse anti-Rabbit IgG (Light Chain specific), HRP Conjugated, S006, 1:5000. CUL4B group, WB: CUL4B rabbit polyclonal antibody, Immunoway, YM5188, 1:1000, 110KD; Second antibody: Mouse anti-Rabbit IgG (Light Chain specific), HRP Conjugated, S006, 1:5000.62KD

To further examine the comprehensive information obtain from the identified protein data, the PPI network was analyzed. The network model was generated using the STRING website. A merged network is shown in (Fig S7 and S8), and, significant proteins annotation (show 50 if available) are shown in (Table S5 and S6). Seven hundred thirty-eight proteins after filter were screened into the PPI network complex, which showed that there were 244 significant enriched interactions among 60 proteins in the group control-A549 (Fig. 7A). Moreover, in the group LPS-A549, it contained 43 significant enriched interactions among 29 proteins (Fig. 7B). Some explanations of protein–protein interaction links are shown (Fig. 7C).

### Ubiquitination and de-ubiquitination enrichment related to OTUB1

In the analysis of protein mass spectrometry, OTUB1(known as a deubiquitinases) is of particular interest. OTUB1 belongs to the ovarian cancer proteases family. In this study, we found that Na, K-ATPase α 1 can bind to the deubiquitinase OTUB1 by protein mass spectrometry. Also, in the A549 cell group, GO analysis of Na, K-ATPase α1 interacting proteins showed significant enrichment of ubiquitination and deubiquitination, both were related to OTUB1. The enrichment items of ubiquitination and deubiquitination enrichment items are shown in (Table 2).

### Na, K-ATPase α 1 interacts with SQSTM1 and CUL4B through Co-IP and western blot verification

Protein ubiquitination is a key step in the ubquitin-proteasome degradation pathway, and autophagy plays an indispensable role in maintaining cell homeostasis, clearing excess proteins and organelles, apoptosis, metabolism and senescence. We next selected the autophagy-related protein (SQSTM1) and the scaffold protein in CUL4B-RING ubiquitin ligase (CRL4B) complexes (CUL4B) from the significantly differentially expressed proteins for verification. Western blot assay followed after the Co-IP by Na, K-ATPase α1 antibody showed that both of SQSTM1 and CUL4B were positive (Fig. 7D), which indicated that there was a close relationship between Na, K-ATPase α1 and proteins related to autophagy and ubiquitination pathway. In all, further studies are needed to verify these results.

## Discussion

Proteomic methods can not only study the whole set of proteins of ARDS, but also verify the drugs that may be effective in the treatment of ARDS. To our knowledge, this study is the first to determine the binding proteins of Na, K-ATPase α1 in ARDS by proteomic technologies from the perspective of alveolar fluid clearance. Our quantitative discovery-based proteomic approach identified commonalities as well as significant differences in the binding proteins of Na, K-ATPase α1 between A549 cells and LPS-induced A549 cells. We utilized PPI network analysis to select PPI and gene co-expression proteins that were linked to Na, K-ATPase α1. Furthermore, we conducted function and pathway analysis to seek biological pathways that may have an impact on ARDS.

We screened these proteins interacted with Na, K-ATPase α1, and carried out the related GO/KEGG analysis. According to the GO analysis, we found that almost all of the most enriched and meaningful BP terms were related to biosynthetic process in the LPS-A549 group. The mainly enriched terms were closely related to enzymatic activity and protein binding. KEGG analysis showed that the proteins were primarily enriched in RNA transport and Fatty acid metabolism. The PPI network was built on the binding proteins that was analyzed by STRING website. We observed that there were 43 significant enriched interactions among 29 proteins in the LPS-A549 group. Besides, we found that there were obvious ubiquitination and deubiquitination phenomena, as well as the pathways related to autophagy.

Based on these results, we chose some proteins with expression levels that were significantly expressed for further verification. Among the most expressed proteins, there were several intriguing proteins, including the deubiquitinase (OTUB1), the tight junction protein zonula occludens-1 (ZO-1), the scaffold protein in CUL4B-RING ubiquitin ligase (CRL4B) complexes (CUL4B) and the autophagy-related protein SQSTM1.

Ubiquitination is a type of protein post-translational modification [14]. Our GO analysis of Na, K-ATPase α1 interaction protein showed that ubiquitination and deubiquitination were significantly enriched, and both were related to OTUB1 (Table 2). OTUB1, known as a deubiquitinases, can protect the protein from degradation and belongs to the ovarian cancer proteases family. Zhang W et al. found that OTUB1 performed as a molecular indicator of poor prognosis in digestive cancers, regulated the infiltration of tumor immunocytes, and exerted a significant influence on apoptosis and autophagy [15]. Our study found that LPS reduced the expression of OTUB1, which may act directly with Na, K-ATPase α1. Therefore, LPS may decrease the level of Na, K-ATPase α1 to lessen its protection by decreasing OTUB1. Combined with the previous conclusion, we speculate that up-regulating OTUB1 can protect Na, K-ATPase α1 from E3 ubiquitin ligase degradation, thus increasing Na, K-ATPase abundance and enzyme activity. More studies are needed to confirm whether OTUB1 can be a therapeutic site for ARDS in the future.

**Table 2:**
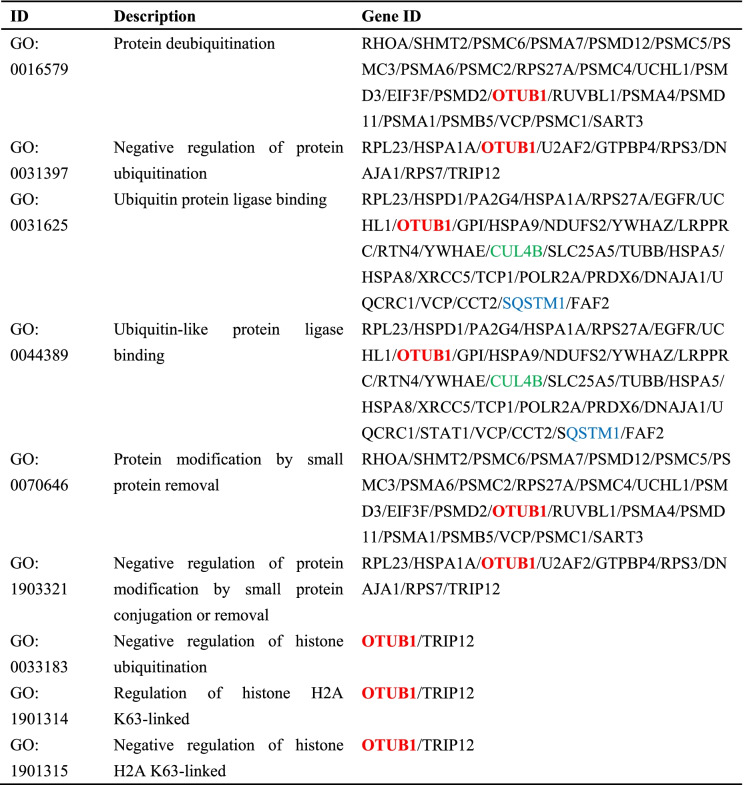
The enrichment items of ubiquitination and deubiquitination by GO analysis with Na, K-ATPase α1 interacting protein

Otub1 is marked in red, CUL4B is marked in green, SQSTM1 is marked in blue

Tight junction is one of the important components of capillary-alveolar barrier, which plays an important role in reducing lung water production and stabilizing lung microenvironment [16]. ZO-1, a tight junction protein, regulates signal transduction, transcription, and cellular communication. The down-regulation of its expression or activity can affect the formation of tight junctions between cells [17, 18]. Ni JJ et al. found that plasma ZO-1 proteins appear to be a valuable prognostic biomarker for the severity of sepsis and a predictor of 30-day mortality for patients with sepsis [19]. And Lee TJ et al. found that ZO-1 on the exotoxin LPS of *P. aeruginosa*-induced diseases could be critical in the development of novel therapeutics [20]. It is interesting that, Na, K-ATPase β1 promotes the expression of key proteins such as ZO-1, ZO-2, occludin and claudin-18 in tight junction complex and reduces the production of lung water [21]. In our study, the level of ZO-1 mRNA in lung tissue of ARDS rats induced by LPS was significantly lower than that in control group [22]. Accordingly, we speculate that increasing the level of Na, K-ATPase α1/β1 may enhance the tight junction of lung epithelium and reduce the production of lung water. The follow-up experiments are needed to verify our theory.

CUL4B, which acts as a scaffold protein in CUL4B-RING ubiquitin ligase (CRL4B) complexes, participates in a variety of biological processes [23]. Song Y et al. reported that CUL4B functions to restrict TLR-triggered inflammatory responses through regulating the AKT-GSK3β pathway [24]. Our proteomic results show that CUL4B may bind to Na, K-ATPase α1 (Fig. 7D), and the therapeutic target site of ARDS may extend to the effect of Na, K-ATPase α1 on CUL4B in subsequent studies.

SQSTM1 is known as an autophagy protein that is involved in ubiquitin–proteasome and autophagy-lysosome degradation processes [25]. Liu Y et al. revealed that the relationship between Na, K-ATPase and autophagy-lysosome pathway requires the involvement of α1 subunit, and Na, K-ATPase α1 and AMPK may act as the “on” and “off” switch of autophagy pathway [26]. More importantly, Na, K-ATPase can be degraded through the ubiquitin–proteasome pathway and the autophagy-lysosome pathway. Autophagy defects can lead to SQSTM1 accumulation and induce cell stress and disease. In our study, we found that Na, K-ATPaseα1 could bind to SQSTM1 by protein profiling, which was verified by endogenous protein interaction analysis (Fig. 7D). Consequently, the decrease of SQSTM1 mRNA expression may be helpful to reduce the transport of polyubiquitinated Na, K-ATPase α1 to autophagy-lysosome system for degradation. The effect of their interaction on the abundance and enzyme activity of Na, K-ATPase α1, the improvement of lung water removal ability of alveolar cells, and the improvement of the prognosis of ARDS is worth extensive attention and discussion in the future.

As we all know, biomarkers are the most direct, rapid and effective diagnostic tools, and their screening and acquisition can play an important role in many aspects of tumor diagnosis, development, treatment, and efficacy monitoring. Consideration these proteins as biomarkers for ARDS have provided valuable insight into the pathogenesis. This is a new hope of identifying new biomarkers for prediction, prognostication, and diagnosis of ARDS. Our study is the first attempt to understand the mechanism of ARDS occurrence by exploring the changes of Na, K-ATPase α1-binding proteins in ARDS, which can serve to find new targets for drug therapy of ARDS.

Our present study and these previous studies suggest that OTUB1, ZO-1, CUL4B and SQSTM1 can act as therapeutic targets for ARDS cases of different etiologies. For these few proteins we identified, we can develop relevant drugs for targeted therapy. These results are preliminary and require a larger sample size for longitudinal studies and a large number of follow-up animal experiments and clinical trials for validation, which leads to the limitation of our study that the ubiquitin-related, autophagy-related and tight junction related proteins we identified are only present in A549 cells.

Nevertheless, most previous studies have been conducted in lung tissue, plasma, bronchoalveolar lavage fluid of ARDS patients, rats and mice [27], and at the cellular level only Janga H [28] revealed factors associated with LPS-mediated lung injury using H441 epithelial cells and endothelial cells under LS-MS based proteomics. We need to design further studies to investigate whether these changes are also present in primary mouse alveolar epithelial cells and in lung tissue, plasma and bronchoalveolar lavage fluid in animal models of ARDS. Also, our study might have been more meaningful if we extend these studies to human cell lines, tissues and subjects, as this would provide direct evidence for the role of these proteins in the development of ARDS in human.

## Conclusion

In summary, using a quantitative discovery-based proteomic approach, this study identified commonalities as well as significant differences in protein expression profiles of ARDS cells model. Notably, the development of ARDS is related to many pathways. We could roughly screen the important proteins and pathways related to the progression of ARDS, and propose possible therapy of extractive proteins including OTUB1, ZO-1, CUL4B and SQSTM1. These key proteins still need to be tested using a large quantity of clinical specimens, and to be analyzed and validated in combination with the individual conditions of clinical patients. Further well-designed studies developing diagnostic panels and therapeutic targets based on these aberrantly expressed proteins and exploring the roles of these proteins that were most beneficial to ARDS are needed.

## Supplementary Information

**Additional file 1:** **Table S1.** Sample grouping. **Table S2.** Experimental results and Statistics. **Table S3.** Summary of significant proteins identified in the study. **Table S4.** Top 20 up-regulated KEGG pathway analysis. **Table S5.** Results of group Control-A549–IgG-A549. Significant proteins annotation (show 50 if available). **Table S6.** Results of group LPS-A549–IgG-LPS. Significant proteins annotation (show 50 if available). **Figure S1.** Venn diagram of the different proteins in LPS-A549 vs. IgG-LPS. **Figure S2.** Venn diagram of the different proteins in control-A549 vs. IgG-A549. **Figure S3.** Enriched GO items of < C > in Control-A549 vs. IgG-A549. top axis is log10(adjust *p*-value), bottom axis is gene count. **Figure S4.** Enriched GO items of < C > in LPS-A549 vs. IgG-LPS. top axis is log10(adjust *p*-value), bottom axis is gene count. **Figure S5.** Enriched KEGG items of < T > in Control-A549 vs. IgG-A549. **Figure S6.** Enriched KEGG items of < T > in LPS-A549 vs. IgG-LPS. **Figure S7.** Control-A549--IgG-A549-STRINGdb-T-1. **Figure S8.** LPS-A549--IgG-LPS-STRINGdb-T-1.

**Additional file 2.**

**Additional file 3.**

**Additional file 4.**

## Data Availability

The datasets supporting the conclusions of this article are included within the article and its additional files.

## Abbreviations

ARDS: Acute respiratory distress syndrome
ALI: Acute lung injury
ICU: Intensive care unit
LC–MS/MS: Liquid chromatography-tandem mass spectrometry
AP-MS/MS: Affinity purification mass spectrometry
Co-IP-MS: Co-immunoprecipitation mass spectrometry
AT II: Alveolar type II epithelial cells
LPS: Lipopolysaccharide
ENaC: Apically-located epithelial Na + channel
A549: Human non-small cell lung cancer cell line/Human AT II cell line
AFC: Alveolar fluid clearance
GO: Gene Ontology
KEGG: Kyoto Encyclopedia of Genes and Genomes
