# Supplementary material for: Identification of different proteins binding to Na, K-ATPase α1 in LPS-induced ARDS cell model by proteomic analysis
Source: Proteome Sci. 2022 Jun 9;20:10. doi: 10.1186/s12953-022-00193-3 (PMC9178877; doi:10.1186/s12953-022-00193-3)
Supplement: Supplementary file 1 — Additional file 1: Table S1. Sample grouping. Table S2. Experimental results and Statistics. Table S3. Summary of significant proteins identified in the study. Table S4. Top 20 up-regulated KEGG pathway analysis. Table S5. Results of group Control-A549–IgG-A549. Significant proteins annotation (show 50 if available). Table S6. Results of group LPS-A549–IgG-LPS. Significant proteins annotation (show 50 if available). Figure S1. Venn diagram of the different proteins in LPS-A549 vs. IgG-LPS. Figure S2. Venn diagram of the different proteins in control-A549 vs. IgG-A549. Figure S3. Enriched GO items of < C > in Control-A549 vs. IgG-A549. top axis is log10(adjust p-value), bottom axis is gene count. Figure S4. Enriched GO items of < C > in LPS-A549 vs. IgG-LPS. top axis is log10(adjust p-value), bottom axis is gene count. Figure S5. Enriched KEGG items of < T > in Control-A549 vs. IgG-A549. Figure S6. Enriched KEGG items of < T > in LPS-A549 vs. IgG-LPS. Figure S7. Control-A549--IgG-A549-STRINGdb-T-1. Figure S8. LPS-A549--IgG-LPS-STRINGdb-T-1. [file 12953_2022_193_MOESM1_ESM.docx]

**Identification of different proteins binding to Na, K-ATPase α1 in lipopolysaccharide-induced acute respiratory distress syndrome cell model by proteomic analysis**

Xu-Peng Wen^1^, Yue-Zhong Zhang^2^, He Huang^3^, Tao-Hua Liu^2^, Qi-Quan Wan^1*^

*Correspondence should be addressed to Qi-Quan Wan; [13548685542@163.com](mailto:13548685542@163.com)

Supplementary Tables: 6

Supplemental Figures: 8

**Supplemental Tables**

Table S1. Sample grouping.

Table S2. Experimental results and Statistics

Table S3. Summary of significant proteins identified in the study

Table S4. Top 20 up-regulated KEGG pathway analysis

Table S5. Results of group Control-A549–IgG-A549. Significant proteins annotation (show 50 if available)

Table S6. Results of group LPS-A549–IgG-LPS. Significant proteins annotation (show 50 if available)

**Supplemental Figures**

Figure S1. Venn diagram of the different proteins in LPS-A549 vs. IgG-LPS

Figure S2. Venn diagram of the different proteins in control-A549 vs. IgG-A549

Figure S3. Enriched GO items of < C > in Control-A549 vs. IgG-A549. top axis is log10(adjust p-value), bottom axis is gene count.

Figure S4. Enriched GO items of < C > in LPS-A549 vs. IgG-LPS. top axis is log10(adjust p-value), bottom axis is gene count.

Figure S5. Enriched KEGG items of < T > in Control-A549 vs. IgG-A549

Figure S6. Enriched KEGG items of < T > in LPS-A549 vs. IgG-LPS

Figure S7. Control-A549--IgG-A549-STRINGdb-T-1

Figure S8. LPS-A549--IgG-LPS-STRINGdb-T-1

**Supplemental Tables**

**Table S1**. Sample grouping.

| Sample ID | Tag | IP Condition |
| --- | --- | --- |
| A549 | IgG | IgG |
|  | A549 | Antibody |
| A549 LPS | IgG | IgG |
|  | LPS | Antibody |

The experiment was divided into two groups: A: A549 cells, B: A549 cells +LPS (1μg/ml, cultured for 12h)

**T****able S2**. Experimental results and Statistics

| Total number of peptides | Total number of protein identified in the experiment | Number of proteins after filtration | Number of differentially expressed proteins |
| --- | --- | --- | --- |
| 10841 | 1598 | 738 | 89 |

**Table S3**. Summary of significant proteins identified in the study

| Group | #Significant |
| --- | --- |
| Control-A549—IgG-A549.T | 698 |
| Control-A549—IgG-A549.C | 22 |
| Control-A549—IgG-A549.T&C | 660 |
| LPS-A549—IgG-LPS.T | 478 |
| LPS-A549—IgG-LPS.C | 17 |
| LPS-A549—IgG-LPS.T&C | 796 |

Note. Test enriched (T), control enriched (C), and none enriched (T&C)

**Table S4**. Top 20 up-regulated KEGG pathway analysis

| Pathway ID | | Definition | Counts | | Q-value | P-value |
| --- | --- | --- | --- | --- | --- | --- |
| hsa03013 | RNA transport | | | 28 | 3.63E-08 | 3.35E-10 |
| hsa01200 | Carbon metabolism | | | 20 | 6.76E-06 | 9.35E-08 |
| hsa03010 | Ribosome | | | 23 | 7.57E-06 | 1.40E-07 |
| hsa01212 | Fatty acid metabolism | | | 13 | 1.01E-05 | 2.57E-07 |
| hsa03050 | Proteasome | | | 12 | 1.01E-05 | 2.78E-07 |
| hsa03008 | Ribosome biogenesis in eukaryotes | | | 18 | 1.41E-05 | 4.55E-07 |
| hsa00010 | Glycolysis / Gluconeogenesis | | | 14 | 2.44E-05 | 8.99E-07 |
| hsa00970 | Aminoacyl-tRNA biosynthesis | | | 13 | 8.99E-05 | 3.75E-06 |
| hsa00620 | Pyruvate metabolism | | | 10 | 8.99E-05 | 4.14E-06 |
| hsa00071 | Fatty acid degradation | | | 10 | 0.000262879 | 1.33E-05 |
| hsa00020 | Citrate cycle (TCA cycle) | | | 8 | 0.000516237 | 2.86E-05 |
| hsa04964 | Proximal tubule bicarbonate reclamation | | | 7 | 0.000596749 | 3.58E-05 |
| hsa01230 | Biosynthesis of amino acids | | | 12 | 0.001215531 | 7.85E-05 |
| hsa04141 | Protein processing in endoplasmic reticulum | | | 19 | 0.001237167 | 8.56E-05 |
| hsa03030 | DNA replication | | | 8 | 0.001594835 | 0.000117677 |
| hsa05230 | Central carbon metabolism in cancer | | | 10 | 0.005471872 | 0.000428984 |
| hsa04961 | "Endocrine and other factor-regulated calcium | | | 8 | 0.009728765 | 0.000807582 |
| hsa00280 | reabsorption" | | | 8 | 0.010651902 | 0.000933334 |
| hsa00670 | Valine, leucine and isoleucine degradation | | | 5 | 0.014429513 | 0.001330877 |
| hsa00030 | One carbon pool by folate | | | 6 | 0.015175363 | 0.001539636 |

**Table S5.**  Results of group Control-A549–IgG-A549

Significant proteins annotation (show 50 if available)

| Symbol | #Unique | FC | Control-  A549 | IgG-A549 | Annotation |
| --- | --- | --- | --- | --- | --- |
| PRKDC | 159 | 6.43 | 200 | 0 | protein kinase, DNA-activated, catalytic polypeptide |
| DYNC1H1 | 126 | 5.46 | 101 | 0 | dynein, cytoplasmic 1, heavy chain 1; Cytoplasmic dynein 1 acts as a motor for the intracellular retrograde motility of vesicles and organelles along microtubules. Dynein has ATPase activity; the force-producing power stroke is thought to occur on release of ADP |
| ATP1A1 | 40 | 5.39 | 96 | 0 | ATPase, Na+/K+ transporting, alpha 1 polypeptide; This is the catalytic component of the active enzyme, which catalyzes the hydrolysis of ATP coupled with the exchange of sodium and potassium ions across the plasma membrane. This action creates the electrochemical gradient of sodium and potassium ions, providing the energy for active transport of various nutrients |
| PRPF8 | 81 | 5.22 | 85 | 0 | PRP8 pre-mRNA processing factor 8 homolog (S. cerevisiae); Functions as a scaffold that mediates the ordered assembly of spliceosomal proteins and snRNAs. Required for the assembly of the U4/U6-U5 tri-snRNP complex. Functions as scaffold that positions spliceosomal U2, U5 and U6 snRNAs at splice sites on pre-mRNA substrates, so that splicing can occur.  Interacts with both the 5’ and the 3’ splice site |
| FASN | 77 | 5.06 | 76 | 0 | fatty acid synthase |
| DDX21 | 35 | 4.64 | 56 | 0 | DEAD (Asp-Glu-Ala-Asp) box helicase 21; Can unwind double-stranded RNA (helicase) and can fold or introduce a secondary structure to a single-stranded RNA (foldase). Functions as cofactor for JUN-activated transcription. Involved in rRNA processing |
| FLNA | 61 | 4.61 | 55 | 0 | ftlamin A, alpha; Promotes orthogonal branching of actin ftlaments and links actin ftlaments to membrane glycoproteins. Anchors various transmembrane proteins to the actin cytoskeleton and serves as a scaffold for a wide range of cytoplasmic signaling proteins.  Interaction with FLNA may allow neuroblast migration from the ventricular zone into the cortical plate.  Tethers cell surface- localized furin, modulates its rate of internalization and directs its intracellular trafficking  (By similarity). Involved in ciliogenesis |
| MYOF | 52 | 4.40 | 47 | 0 | myoferlin |
| ILF3 | 32 | 4.37 | 46 | 0 | interleukin enhancer binding factor 3, 90kDa |
| EEF2 | 39 | 4.37 | 46 | 0 | eukaryotic translation elongation factor 2; Catalyzes the GTP-dependent ribosomal translocation step during translation elongation. During this step, the ribosome changes from the pre-translocational (PRE) to the post- translocational (POST) state as the newly formed A-site-bound peptidyl-tRNA and P-site-bound deacylated tRNA move to the P and E sites, respectively. Catalyzes the coordinated movement of the two tRNA molecules, the mRNA and conformational changes in the ribosome |
| MYO1C | 41 | 4.31 | 44 | 0 | myosin IC; Myosins are actin-based motor molecules with ATPase activity. Unconventional myosins serve in intracellular movements. Their highly divergent tails are presumed to bind to membranous compartments, which would be moved relative to actin ftlaments.  Involved in glucose transporter recycling in response to insulin by regulating movement of intracellular  GLUT4-containing vesicles to the plasma membrane. Component of the hair cell’s (the sensory cells of the inner ear) adaptation-motor complex. Acts as a mediator of adaptation of mechanoelectrical  transduction in stereocilia of ve [...] |

| ACLY | 39 | 4.28 | 43 | 0 | ATP citrate lyase; ATP citrate-lyase is the primary enzyme responsible for the synthesis of cytosolic acetyl-CoA in many tissues. Has a central role in de novo lipid synthesis. In nervous tissue it may be involved in the biosynthesis of acetylcholine |
| --- | --- | --- | --- | --- | --- |
| FLNB | 53 | 4.21 | 41 | 0 | ftlamin B, beta |
| SNRNP200 | 50 | 4.21 | 41 | 0 | small nuclear ribonucleoprotein 200kDa (U5); RNA helicase that plays an essential role in pre-mRNA splicing as component of the U5 snRNP and U4/U6-U5 tri-snRNP complexes. Involved in spliceosome assembly, activation and disassembly. Mediates changes in the dynamic network of RNA-RNA interactions in the spliceosome. Catalyzes the ATP-dependent unwinding of U4/U6 RNA duplices, an essential step in the assembly of a catalytically active spliceosome |
| CLTC | 72 | 4.21 | 84 | 1 | clathrin, heavy chain (Hc); Clathrin is the major protein of the polyhedral coat of coated pits and vesicles. Two different adapter protein complexes link the clathrin lattice either to the plasma membrane or to the trans-Golgi network |
| TJP1 | 36 | 4.07 | 37 | 0 | tight junction protein 1; The N-terminal may be involved in transducing a signal required for tight junction assembly, while the C-terminal may have speciftc properties of tight junctions. The alpha domain might be involved in stabilizing junctions. Plays a role in the regulation of cell migration by targeting CDC42BPB to the leading edge of migrating cells |
| PGD | 20 | 4.07 | 37 | 0 | phosphogluconate dehydrogenase; Catalyzes the oxidative decarboxylation of 6- phosphogluconate to ribulose 5-phosphate and CO(2), with concomitant reduction of NADP to NADPH (By similarity) |
| NOP56 | 25 | 4.03 | 36 | 0 | NOP56 ribonucleoprotein homolog (yeast); Involved in the early to middle stages of 60S ribosomal subunit biogenesis |
| MATR3 | 26 | 4.00 | 35 | 0 | matrin 3; May play a role in transcription or may interact with other nuclear matrix proteins to form the internal ftbrogranular network. In association with the SFPQ-NONO heteromer may play a role in nuclear retention of defective RNAs |
| RSL1D1 | 29 | 4.00 | 35 | 0 | ribosomal L1 domain containing 1 |
| H2AFY | 16 | 3.96 | 34 | 0 | H2A histone family, member Y; Variant histone H2A which replaces conventional H2A in a subset of nucleosomes where it represses transcription.  Nucleosomes wrap and compact DNA into chromatin, limiting DNA accessibility to the cellular machineries which require DNA as a template. Histones thereby play a central role in transcription regulation, DNA repair, DNA replication and chromosomal stability.  DNA accessibility is regulated via a complex set of  post- translational modiftcations of histones, also called histone code, and nucleosome remodeling. Involved in  stable X chromosome inactiv [...] |
| HNRNPM | 28 | 3.96 | 34 | 0 | heterogeneous nuclear ribonucleoprotein M; Pre-mRNA binding protein in vivo, binds avidly to poly(G) and poly(U) RNA homopolymers in vitro. Involved in splicing. Acts as a receptor for carcinoembryonic antigen in Kupffer cells, may initiate a series of signaling events leading to tyrosine phosphorylation of proteins and induction of IL-1 alpha, IL-6, IL-10 and tumor necrosis factor alpha cytokines |
| MYH9 | 59 | 3.92 | 33 | 0 | myosin, heavy chain 9, non-muscle; Cellular myosin that appears to play a role in cytokinesis, cell shape, and specialized functions such as secretion and capping |
| MYO1B | 29 | 3.83 | 31 | 0 | myosin IB; Motor protein that may participate in process critical to neuronal development and function such as cell migration, neurite outgrowth and vesicular transport (By similarity) |

| IQGAP1 | 36 | 3.79 | 30 | 0 | IQ motif containing GTPase activating protein 1; Binds |
| --- | --- | --- | --- | --- | --- |
|  |  |  |  |  | to activated CDC42 but does not stimulate its GTPase |
|  |  |  |  |  | activity. It associates with calmodulin. Could serve as |
|  |  |  |  |  | an assembly scaffold for the organization of a |
|  |  |  |  |  | multimolecular complex that would interface incoming |
|  |  |  |  |  | signals to the reorganization of the actin cytoskeleton |
|  |  |  |  |  | at the plasma membrane. May promote neurite |
|  |  |  |  |  | outgrowth |
| RRP12 | 28 | 3.79 | 30 | 0 | ribosomal RNA processing 12 homolog (S. cerevisiae) |
| XRCC5 | 27 | 3.79 | 30 | 0 | X-ray repair complementing defective repair in Chinese |
|  |  |  |  |  | hamster cells 5 (double-strand-break rejoining); Single |
|  |  |  |  |  | stranded DNA-dependent ATP-dependent helicase. Has |
|  |  |  |  |  | a role in chromosome translocation. The DNA helicase |
|  |  |  |  |  | II complex binds preferentially to fork-like ends of |
|  |  |  |  |  | double-stranded DNA in a cell cycle-dependent manner. |
|  |  |  |  |  | It works in the 3’-5’ direction. Binding to DNA may be |
|  |  |  |  |  | mediated by XRCC6. Involved in DNA non-homologous |
|  |  |  |  |  | end joining (NHEJ) required for double-strand break |
|  |  |  |  |  | repair and V(D)J recombination. The XRCC5/6 dimer |
|  |  |  |  |  | acts as regulatory subunit of the DNA-dependent |
|  |  |  |  |  | protein kinase [...] |
| CAD | 36 | 3.74 | 29 | 0 | carbamoyl-phosphate synthetase 2, aspartate |
|  |  |  |  |  | transcarbamylase, and dihydroorotase; This protein is a |
|  |  |  |  |  | ”fusion” protein encoding four enzymatic activities of |
|  |  |  |  |  | the pyrimidine pathway (GATase, CPSase, ATCase and |
|  |  |  |  |  | DHOase) |
| DDX3X | 25 | 3.70 | 28 | 0 | DEAD (Asp-Glu-Ala-Asp) box polypeptide 3, X-linked; |
|  |  |  |  |  | Multifunctional ATP-dependent RNA helicase. The |
|  |  |  |  |  | ATPase activity can be stimulated by various ribo- and |
|  |  |  |  |  | deoxynucleic acids indicative for a relaxed substrate |
|  |  |  |  |  | speciftcity. In vitro can unwind partially double |
|  |  |  |  |  | stranded DNA with a preference for 5’- single stranded |
|  |  |  |  |  | DNA overhangs. Is involved in several steps of gene |
|  |  |  |  |  | expression, such as transcription, mRNA maturation, |
|  |  |  |  |  | mRNA export and translation. However, the exact |
|  |  |  |  |  | mechanisms are not known and some functions may be |
|  |  |  |  |  | speciftc for a subset of mRNAs. Involved in |
|  |  |  |  |  | transcriptional regulation. Can [...] |
| COPA | 34 | 3.70 | 28 | 0 | coatomer protein complex, subunit alpha; The |
|  |  |  |  |  | coatomer is a cytosolic protein complex that binds to |
|  |  |  |  |  | dilysine motifs and reversibly associates with Golgi |
|  |  |  |  |  | non- clathrin-coated vesicles, which further mediate |
|  |  |  |  |  | biosynthetic protein transport from the ER, via the |
|  |  |  |  |  | Golgi up to the trans Golgi network. Coatomer |
|  |  |  |  |  | complex is required for budding from Golgi membranes, |
|  |  |  |  |  | and is essential for the retrograde Golgi-to-ER |
|  |  |  |  |  | transport of dilysine-tagged proteins. In mammals, the |
|  |  |  |  |  | coatomer can only be recruited by membranes |
|  |  |  |  |  | associated to ADP-ribosylation factors (ARFs), which |
|  |  |  |  |  | are small GTP-binding proteins; the [...] |
| EL52 | 19 | 3.65 | 27 | 0 | heat shock protein 90kDa alpha (cytosolic), class A |
|  |  |  |  |  | member 1; Molecular chaperone that promotes the |
|  |  |  |  |  | maturation, structural maintenance and proper |
|  |  |  |  |  | regulation of speciftc target proteins involved for |
|  |  |  |  |  | instance in cell cycle control and signal transduction. |
|  |  |  |  |  | Undergoes a functional cycle that is linked to its |
|  |  |  |  |  | ATPase activity. This cycle probably induces |
|  |  |  |  |  | conformational changes in the client proteins, thereby |
|  |  |  |  |  | causing their activation. Interacts dynamically with |
|  |  |  |  |  | various co-chaperones that modulate its substrate |
|  |  |  |  |  | recognition, ATPase cycle and chaperone function |
| ATP1A3 | 17 | 3.60 | 26 | 0 | ATPase, Na+/K+ transporting, alpha 3 polypeptide; |
|  |  |  |  |  | This is the catalytic component of the active enzyme, |
|  |  |  |  |  | which catalyzes the hydrolysis of ATP coupled with the |
|  |  |  |  |  | exchange of sodium and potassium ions across the |
|  |  |  |  |  | plasma membrane. This action creates the |
|  |  |  |  |  | electrochemical gradient of sodium and potassium ions, |
|  |  |  |  |  | providing the energy for active transport of various |
|  |  |  |  |  | nutrients |

| ATP1B1 | 15 | 3.60 | 26 | 0 | ATPase, Na+/K+ transporting, beta 1 polypeptide; This is the non-catalytic component of the active enzyme, which catalyzes the hydrolysis of ATP coupled with the exchange of Na(+) and K(+) ions across the plasma membrane. The beta subunit regulates, through assembly of alpha/beta heterodimers, the number of sodium pumps transported to the plasma membrane |
| --- | --- | --- | --- | --- | --- |
| NOP2 | 23 | 3.60 | 26 | 0 | NOP2 nucleolar protein homolog (yeast); May play a role in the regulation of the cell cycle and the increased nucleolar activity that is associated with the cell proliferation. May act as ribosomal RNA methyltransferase |
| CPS1 | 30 | 3.55 | 25 | 0 | carbamoyl-phosphate synthase 1, mitochondrial; Involved in the urea cycle of ureotelic animals where the enzyme plays an important role in removing excess ammonia from the cell |
| HADHA | 24 | 3.55 | 25 | 0 | hydroxyacyl-CoA dehydrogenase/3-ketoacyl-CoA thiolase/enoyl-CoA hydratase (trifunctional protein), alpha subunit; Bifunctional subunit |
| PDCD11 | 26 | 3.55 | 25 | 0 | programmed cell death 11; Essential for the generation of mature 18S rRNA, speciftcally necessary for cleavages at sites A0, 1 and 2 of the 47S precursor.  Directly interacts with U3 snoRNA |
| RPN1 | 21 | 3.49 | 24 | 0 | ribophorin I; Essential subunit of the N-oligosaccharyl transferase (OST) complex which catalyzes the transfer of a high mannose oligosaccharide from a lipid-linked oligosaccharide donor to an asparagine residue within an Asn-X-Ser/Thr consensus motif in nascent polypeptide chains |
| HADHB | 24 | 3.49 | 24 | 0 | hydroxyacyl-CoA dehydrogenase/3-ketoacyl-CoA thiolase/enoyl-CoA hydratase (trifunctional protein), beta subunit |
| SRRM2 | 24 | 3.44 | 23 | 0 | serine/arginine repetitive matrix 2 |
| DDX27 | 20 | 3.44 | 23 | 0 | DEAD (Asp-Glu-Ala-Asp) box polypeptide 27 |
| SMARCA5 | 24 | 3.44 | 23 | 0 | SWI/SNF related, matrix associated, actin dependent regulator of chromatin, subfamily a, member 5; Helicase that possesses intrinsic ATP-dependent nucleosome-remodeling activity. Complexes containing SMARCA5 are capable of forming ordered nucleosome arrays on chromatin; this may require intact histone H4 tails. Also required for replication of pericentric heterochromatin in S-phase speciftcally in conjunction with BAZ1A. Probably plays a role in repression of polI dependent transcription of the rDNA locus, through the recruitment of the SIN3/HDAC1 corepressor complex to the rDNA promot [...] |
| DHX15 | 23 | 3.44 | 23 | 0 | DEAH (Asp-Glu-Ala-His) box polypeptide 15;  Pre-mRNA processing factor involved in disassembly of spliceosomes after the release of mature mRNA (By  similarity) |
| BRIX1 | 16 | 3.44 | 23 | 0 | BRX1, biogenesis of ribosomes, homolog (S. cerevisiae); Required for biogenesis of the 60S ribosomal subunit |
| DHX9 | 50 | 3.40 | 72 | 2 | DEAH (Asp-Glu-Ala-His) box polypeptide 9; Unwinds double-stranded DNA and RNA in a 3’ to 5’ direction. Alteration of secondary structure may subsequently influence interactions with proteins or other nucleic acids. Functions as a transcriptional activator.  Component of the CRD- mediated complex that promotes MYC mRNA stability. Involved with LARP6 in the stabilization of type I collagen mRNAs for CO1A1 and CO1A2. Positively regulates HIV-1  LTR-directed gene expression |

| HEL-S-123m | 29 | 3.40 | 47 | 1 | ATP synthase, H+ transporting, mitochondrial F1 complex, alpha subunit 1, cardiac muscle; Mitochondrial membrane ATP synthase (F(1)F(0) ATP synthase or Complex V) produces ATP from ADP in the presence of a proton gradient across the membrane which is generated by electron transport complexes of the respiratory chain. F-type ATPases consist of two structural domains, F(1) - containing the extramembraneous catalytic core, and F(0) - containing the membrane proton channel, linked together by a central stalk and a peripheral stalk. During catalysis, ATP synthesis in the catalytic domain of [...] |
| --- | --- | --- | --- | --- | --- |
| UGDH | 20 | 3.38 | 22 | 0 | UDP-glucose 6-dehydrogenase; Involved in the biosynthesis of glycosaminoglycans; hyaluronan, chondroitin sulfate, and heparan sulfate |
| HEL-S-53e | 18 | 3.38 | 22 | 0 | aldehyde dehydrogenase 1 family, member A1; Binds free retinal and cellular retinol-binding protein- bound retinal. Can convert/oxidize retinaldehyde to retinoic acid (By similarity) |
| HNRNPUL2 | 20 | 3.38 | 22 | 0 | heterogeneous nuclear ribonucleoprotein U-like 2 |
| XRCC6 | 17 | 3.32 | 21 | 0 | X-ray repair complementing defective repair in Chinese hamster cells 6; Single stranded DNA-dependent  ATP-dependent helicase. Has a role in chromosome translocation. The DNA helicase II complex binds preferentially to fork-like ends of double-stranded DNA in a cell cycle-dependent manner. It works in the 3’-5’ direction. Binding to DNA may be mediated by XRCC6. Involved in DNA non-homologous end joining (NHEJ) required for double-strand break repair and V(D)J recombination. The XRCC5/6 dimer acts as regulatory subunit of the DNA-dependent protein  kinase complex DNA-PK by increasing the [...] |

**Table S6**. Results of group LPS-A549–IgG-LPS

Significant proteins annotation (show 50 if available)

| Symbol | #Unique | FC | LPS-A549 | IgG-LPS | Annotation |
| --- | --- | --- | --- | --- | --- |
| DYNC1H1 | 126 | 5.03 | 103 | 0 | dynein, cytoplasmic 1, heavy chain 1; Cytoplasmic |
|  |  |  |  |  | dynein 1 acts as a motor for the intracellular retrograde |
|  |  |  |  |  | motility of vesicles and organelles along microtubules. |
|  |  |  |  |  | Dynein has ATPase activity; the force-producing power |
|  |  |  |  |  | stroke is thought to occur on release of ADP |
| PRPF8 | 81 | 4.64 | 78 | 0 | PRP8 pre-mRNA processing factor 8 homolog (S. |
|  |  |  |  |  | cerevisiae); Functions as a scaffold that mediates the |
|  |  |  |  |  | ordered assembly of spliceosomal proteins and snRNAs. |
|  |  |  |  |  | Required for the assembly of the U4/U6-U5 tri-snRNP |
|  |  |  |  |  | complex. Functions as scaffold that positions |
|  |  |  |  |  | spliceosomal U2, U5 and U6 snRNAs at splice sites on |
|  |  |  |  |  | pre-mRNA substrates, so that splicing can occur. |
|  |  |  |  |  | Interacts with both the 5’ and the 3’ splice site |
| FASN | 77 | 4.43 | 67 | 0 | fatty acid synthase |
| FLNA | 61 | 4.28 | 60 | 0 | ftlamin A, alpha; Promotes orthogonal branching of |
|  |  |  |  |  | actin ftlaments and links actin ftlaments to membrane |
|  |  |  |  |  | glycoproteins. Anchors various transmembrane proteins |
|  |  |  |  |  | to the actin cytoskeleton and serves as a scaffold for a |
|  |  |  |  |  | wide range of cytoplasmic signaling proteins. |
|  |  |  |  |  | Interaction with FLNA may allow neuroblast migration |
|  |  |  |  |  | from the ventricular zone into the cortical plate. |
|  |  |  |  |  | Tethers cell surface- localized furin, modulates its rate |
|  |  |  |  |  | of internalization and directs its intracellular trafficking |
|  |  |  |  |  | (By similarity). Involved in ciliogenesis |
| FLNB | 53 | 4.06 | 51 | 0 | ftlamin B, beta |
| EEF2 | 39 | 4.03 | 50 | 0 | eukaryotic translation elongation factor 2; Catalyzes |
|  |  |  |  |  | the GTP-dependent ribosomal translocation step |
|  |  |  |  |  | during translation elongation. During this step, the |
|  |  |  |  |  | ribosome changes from the pre-translocational (PRE) |
|  |  |  |  |  | to the post- translocational (POST) state as the newly |
|  |  |  |  |  | formed A-site-bound peptidyl-tRNA and P-site-bound |
|  |  |  |  |  | deacylated tRNA move to the P and E sites, |
|  |  |  |  |  | respectively. Catalyzes the coordinated movement of |
|  |  |  |  |  | the two tRNA molecules, the mRNA and |
|  |  |  |  |  | conformational changes in the ribosome |
| TJP1 | 36 | 3.89 | 45 | 0 | tight junction protein 1; The N-terminal may be |
|  |  |  |  |  | involved in transducing a signal required for tight |
|  |  |  |  |  | junction assembly, while the C-terminal may have |
|  |  |  |  |  | speciftc properties of tight junctions. The alpha domain |
|  |  |  |  |  | might be involved in stabilizing junctions. Plays a role |
|  |  |  |  |  | in the regulation of cell migration by targeting |
|  |  |  |  |  | CDC42BPB to the leading edge of migrating cells |
| PRKDC | 159 | 3.83 | 136 | 2 | protein kinase, DNA-activated, catalytic polypeptide |
| MYOF | 52 | 3.83 | 43 | 0 | myoferlin |
| SNRNP200 | 50 | 3.63 | 37 | 0 | small nuclear ribonucleoprotein 200kDa (U5); RNA |
|  |  |  |  |  | helicase that plays an essential role in pre-mRNA |
|  |  |  |  |  | splicing as component of the U5 snRNP and U4/U6-U5 |
|  |  |  |  |  | tri-snRNP complexes. Involved in spliceosome |
|  |  |  |  |  | assembly, activation and disassembly. Mediates changes |
|  |  |  |  |  | in the dynamic network of RNA-RNA interactions in |
|  |  |  |  |  | the spliceosome. Catalyzes the ATP-dependent |
|  |  |  |  |  | unwinding of U4/U6 RNA duplices, an essential step in |
|  |  |  |  |  | the assembly of a catalytically active spliceosome |
| IQGAP1 | 36 | 3.55 | 35 | 0 | IQ motif containing GTPase activating protein 1; Binds |
|  |  |  |  |  | to activated CDC42 but does not stimulate its GTPase |
|  |  |  |  |  | activity. It associates with calmodulin. Could serve as |
|  |  |  |  |  | an assembly scaffold for the organization of a |
|  |  |  |  |  | multimolecular complex that would interface incoming |
|  |  |  |  |  | signals to the reorganization of the actin cytoskeleton |
|  |  |  |  |  | at the plasma membrane. May promote neurite |
|  |  |  |  |  | outgrowth |

| PGD | 20 | 3.55 | 35 | 0 | phosphogluconate dehydrogenase; Catalyzes the oxidative decarboxylation of 6- phosphogluconate to ribulose 5-phosphate and CO(2), with concomitant reduction of NADP to NADPH (By similarity) |
| --- | --- | --- | --- | --- | --- |
| MYO1C | 41 | 3.51 | 34 | 0 | myosin IC; Myosins are actin-based motor molecules with ATPase activity. Unconventional myosins serve in intracellular movements. Their highly divergent tails are presumed to bind to membranous compartments, which would be moved relative to actin ftlaments.  Involved in glucose transporter recycling in response to insulin by regulating movement of intracellular  GLUT4-containing vesicles to the plasma membrane. Component of the hair cell’s (the sensory cells of the inner ear) adaptation-motor complex. Acts as a mediator of adaptation of mechanoelectrical  transduction in stereocilia of ve [...] |
| ATP1A1 | 40 | 3.33 | 95 | 2 | ATPase, Na+/K+ transporting, alpha 1 polypeptide; This is the catalytic component of the active enzyme, which catalyzes the hydrolysis of ATP coupled with the exchange of sodium and potassium ions across the plasma membrane. This action creates the electrochemical gradient of sodium and potassium ions, providing the energy for active transport of various nutrients |
| MATR3 | 26 | 3.31 | 29 | 0 | matrin 3; May play a role in transcription or may interact with other nuclear matrix proteins to form the internal ftbrogranular network. In association with the SFPQ-NONO heteromer may play a role in nuclear retention of defective RNAs |
| XRCC5 | 27 | 3.31 | 29 | 0 | X-ray repair complementing defective repair in Chinese hamster cells 5 (double-strand-break rejoining); Single stranded DNA-dependent ATP-dependent helicase. Has a role in chromosome translocation. The DNA helicase II complex binds preferentially to fork-like ends of double-stranded DNA in a cell cycle-dependent manner. It works in the 3’-5’ direction. Binding to DNA may be mediated by XRCC6. Involved in DNA non-homologous end joining (NHEJ) required for double-strand break repair and V(D)J recombination. The XRCC5/6 dimer acts as regulatory subunit of the DNA-dependent protein kinase [...] |
| LRPPRC | 33 | 3.26 | 28 | 0 | leucine-rich pentatricopeptide repeat containing; May play a role in RNA metabolism in both nuclei and mitochondria. In the nucleus binds to  HNRPA1-associated poly(A) mRNAs and is part of nmRNP complexes at late stages of mRNA maturation which are possibly associated with nuclear mRNA export. May bind mature mRNA in the nucleus outer membrane. In mitochondria binds to poly(A) mRNA. Plays a role in translation or stability of mitochondrially encoded cytochrome c oxidase (COX) subunits. May be involved in transcription regulation. Cooperates with PPARGC1A to regulate certain  mitochondria [...] |
| HEL-S-89n | 21 | 3.21 | 27 | 0 | heat shock 70kDa protein 5 (glucose-regulated protein, 78kDa); Probably plays a role in facilitating the assembly of multimeric protein complexes inside the ER |
| eIF3a | 27 | 3.17 | 26 | 0 | eukaryotic translation initiation factor 3, subunit A; Component of the eukaryotic translation initiation factor 3 (eIF-3) complex, which is required for several steps in the initiation of protein synthesis. The eIF-3 complex associates with the 40S ribosome and facilitates the recruitment of eIF-1, eIF-1A,  eIF-2:GTP:methionyl-tRNAi and eIF-5 to form the 43S preinitiation complex (43S PIC). The eIF-3 complex stimulates mRNA recruitment to the 43S PIC and scanning of the mRNA for AUG recognition. The eIF-3 complex is also required for disassembly and recycling of post-termination riboso [...] |

| COPA | 34 | 3.17 | 26 | 0 | coatomer protein complex, subunit alpha; The coatomer is a cytosolic protein complex that binds to dilysine motifs and reversibly associates with Golgi non- clathrin-coated vesicles, which further mediate biosynthetic protein transport from the ER, via the Golgi up to the trans Golgi network. Coatomer  complex is required for budding from Golgi membranes, and is essential for the retrograde Golgi-to-ER transport of dilysine-tagged proteins. In mammals, the coatomer can only be recruited by membranes associated to ADP-ribosylation factors (ARFs), which are small GTP-binding proteins; the [...] |
| --- | --- | --- | --- | --- | --- |
| CPS1 | 30 | 3.12 | 25 | 0 | carbamoyl-phosphate synthase 1, mitochondrial; Involved in the urea cycle of ureotelic animals where the enzyme plays an important role in removing excess ammonia from the cell |
| PABPC1 | 15 | 3.12 | 25 | 0 | poly(A) binding protein, cytoplasmic 1; Binds the poly(A) tail of mRNA. May be involved in cytoplasmic regulatory processes of mRNA metabolism such as pre- mRNA splicing. Its function in translational initiation regulation can either be enhanced by PAIP1 or repressed by PAIP2. Can probably bind to cytoplasmic RNA sequences other than poly(A) in vivo. Involved in translationally coupled mRNA turnover. Implicated with other RNA-binding proteins in the cytoplasmic deadenylation/translational and decay interplay of the FOS mRNA mediated by the major coding-region determinant of instability [...] |
| MYO1B | 29 | 3.12 | 25 | 0 | myosin IB; Motor protein that may participate in process critical to neuronal development and function such as cell migration, neurite outgrowth and vesicular transport (By similarity) |
| SLC3A2 | 21 | 3.06 | 24 | 0 | solute carrier family 3 (activators of dibasic and neutral amino acid transport), member 2; Required for the function of light chain amino-acid transporters.  Involved in sodium-independent, high-affinity transport of large neutral amino acids such as phenylalanine, tyrosine, leucine, arginine and tryptophan. Involved in guiding and targeting of LAT1 and LAT2 to the plasma membrane. When associated with SLC7A6 or SLC7A7 acts as an arginine/glutamine exchanger, following an antiport mechanism for amino acid transport, influencing arginine release in exchange for  extracellular amino acids [...] |
| HEL107 | 20 | 3.06 | 24 | 0 | transketolase; Catalyzes the transfer of a two-carbon ketol group from a ketose donor to an aldose acceptor, via a covalent intermediate with the cofactor thiamine pyrophosphate |
| ILF3 | 32 | 3.06 | 51 | 1 | interleukin enhancer binding factor 3, 90kDa |
| DDX21 | 35 | 3.03 | 50 | 1 | DEAD (Asp-Glu-Ala-Asp) box helicase 21; Can unwind double-stranded RNA (helicase) and can fold or introduce a secondary structure to a single-stranded RNA (foldase). Functions as cofactor for JUN-activated transcription. Involved in rRNA processing |
| ATP2A2 | 23 | 3.01 | 23 | 0 | ATPase, Ca++ transporting, cardiac muscle, slow twitch 2; This magnesium-dependent enzyme catalyzes the hydrolysis of ATP coupled with the translocation of calcium from the cytosol to the sarcoplasmic reticulum lumen. Isoform 2 is involved in the regulation of the contraction/relaxation cycle |
| EL52 | 19 | 3.01 | 23 | 0 | heat shock protein 90kDa alpha (cytosolic), class A member 1; Molecular chaperone that promotes the maturation, structural maintenance and proper regulation of speciftc target proteins involved for instance in cell cycle control and signal transduction. Undergoes a functional cycle that is linked to its ATPase activity. This cycle probably induces conformational changes in the client proteins, thereby causing their activation. Interacts dynamically with various co-chaperones that modulate its substrate recognition, ATPase cycle and chaperone function |

| SF3B1 | 28 | 3.01 | 23 | 0 | splicing factor 3b, subunit 1, 155kDa; Subunit of the splicing factor SF3B required for ’A’ complex assembly formed by the stable binding of U2 snRNP to the branchpoint sequence (BPS) in pre-mRNA. Sequence independent binding of SF3A/SF3B complex upstream of the branch site is essential, it may anchor U2 snRNP to the pre-mRNA. May also be involved in the assembly of the ’E’ complex. Belongs also to the minor U12-dependent spliceosome, which is involved in the splicing of rare class of nuclear pre-mRNA intron |
| --- | --- | --- | --- | --- | --- |
| RPN1 | 21 | 3.01 | 23 | 0 | ribophorin I; Essential subunit of the N-oligosaccharyl transferase (OST) complex which catalyzes the transfer of a high mannose oligosaccharide from a lipid-linked oligosaccharide donor to an asparagine residue within an Asn-X-Ser/Thr consensus motif in nascent polypeptide chains |
| PBEF1 | 19 | 2.95 | 22 | 0 | nicotinamide phosphoribosyltransferase; Catalyzes the condensation of nicotinamide with 5-  phosphoribosyl-1-pyrophosphate to yield nicotinamide mononucleotide, an intermediate in the biosynthesis of NAD. It is the rate limiting component in the  mammalian NAD biosynthesis pathway (By similarity) |
| TRA1 | 19 | 2.95 | 22 | 0 | heat shock protein 90kDa beta (Grp94), member 1; Molecular chaperone that functions in the processing and transport of secreted proteins. When associated with CNPY3, required for proper folding of Toll-like receptors (By similarity). Functions in endoplasmic reticulum associated degradation (ERAD). Has ATPase activity |
| FBL | 14 | 2.95 | 22 | 0 | ftbrillarin; Involved in pre-rRNA processing. Utilizes the methyl donor S-adenosyl-L-methionine to catalyze the site-speciftc 2’- hydroxyl methylation of ribose moieties in pre-ribosomal RNA. Site speciftcity is provided by a guide RNA that base pairs with the substrate. Methylation occurs at a characteristic distance from the sequence involved in base pairing with the guide RNA |
| UQCRC2 | 14 | 2.95 | 22 | 0 | ubiquinol-cytochrome c reductase core protein II; This is a component of the ubiquinol-cytochrome c reductase complex (complex III or cytochrome b-c1 complex), which is part of the mitochondrial respiratory chain.  The core protein 2 is required for the assembly of the complex |
| PFKP | 22 | 2.95 | 22 | 0 | phosphofructokinase, platelet; Catalyzes the third step of glycolysis, the phosphorylation of  fructose-6-phosphate (F6P) by ATP to generate  fructose-1,6-bisphosphate (FBP) and ADP |
| ASPH | 23 | 2.95 | 22 | 0 | aspartate beta-hydroxylase |
| NOP58 | 20 | 2.90 | 21 | 0 | NOP58 ribonucleoprotein homolog (yeast); Required for 60S ribosomal subunit biogenesis (By similarity) |
| XRCC6 | 17 | 2.84 | 20 | 0 | X-ray repair complementing defective repair in Chinese hamster cells 6; Single stranded DNA-dependent  ATP-dependent helicase. Has a role in chromosome translocation. The DNA helicase II complex binds preferentially to fork-like ends of double-stranded DNA in a cell cycle-dependent manner. It works in the 3’-5’ direction. Binding to DNA may be mediated by XRCC6. Involved in DNA non-homologous end joining (NHEJ) required for double-strand break repair and V(D)J recombination. The XRCC5/6 dimer acts as regulatory subunit of the DNA-dependent protein  kinase complex DNA-PK by increasing the [...] |
| ATP1A3 | 17 | 2.84 | 20 | 0 | ATPase, Na+/K+ transporting, alpha 3 polypeptide; This is the catalytic component of the active enzyme, which catalyzes the hydrolysis of ATP coupled with the exchange of sodium and potassium ions across the plasma membrane. This action creates the electrochemical gradient of sodium and potassium ions, providing the energy for active transport of various nutrients |
| RRP12 | 28 | 2.84 | 20 | 0 | ribosomal RNA processing 12 homolog (S. cerevisiae) |

| CCT3 | 18 | 2.77 | 19 | 0 | chaperonin containing TCP1, subunit 3 (gamma); |
| --- | --- | --- | --- | --- | --- |
|  |  |  |  |  | Molecular chaperone; assists the folding of proteins |
|  |  |  |  |  | upon ATP hydrolysis. As part of the BBS/CCT |
|  |  |  |  |  | complex may play a role in the assembly of BBSome, a |
|  |  |  |  |  | complex involved in ciliogenesis regulating transports |
|  |  |  |  |  | vesicles to the cilia. Known to play a role, in vitro, in |
|  |  |  |  |  | the folding of actin and tubulin |
| CAD | 36 | 2.77 | 19 | 0 | carbamoyl-phosphate synthetase 2, aspartate |
|  |  |  |  |  | transcarbamylase, and dihydroorotase; This protein is a |
|  |  |  |  |  | ”fusion” protein encoding four enzymatic activities of |
|  |  |  |  |  | the pyrimidine pathway (GATase, CPSase, ATCase and |
|  |  |  |  |  | DHOase) |
| EPRS | 26 | 2.77 | 19 | 0 | glutamyl-prolyl-tRNA synthetase |
| ACLY | 39 | 2.76 | 41 | 1 | ATP citrate lyase; ATP citrate-lyase is the primary |
|  |  |  |  |  | enzyme responsible for the synthesis of cytosolic |
|  |  |  |  |  | acetyl-CoA in many tissues. Has a central role in de |
|  |  |  |  |  | novo lipid synthesis. In nervous tissue it may be |
|  |  |  |  |  | involved in the biosynthesis of acetylcholine |
| BRIX1 | 16 | 2.71 | 18 | 0 | BRX1, biogenesis of ribosomes, homolog (S. cerevisiae); |
|  |  |  |  |  | Required for biogenesis of the 60S ribosomal subunit |
| DHX15 | 23 | 2.64 | 17 | 0 | DEAH (Asp-Glu-Ala-His) box polypeptide 15; |
|  |  |  |  |  | Pre-mRNA processing factor involved in disassembly of |
|  |  |  |  |  | spliceosomes after the release of mature mRNA (By |
|  |  |  |  |  | similarity) |
| G6PD | 29 | 2.63 | 37 | 1 | glucose-6-phosphate dehydrogenase; Produces pentose |
|  |  |  |  |  | sugars for nucleic acid synthesis and main producer of |
|  |  |  |  |  | NADPH reducing power |
| PTBP1 | 12 | 2.56 | 16 | 0 | polypyrimidine tract binding protein 1; Plays a role in |
|  |  |  |  |  | pre-mRNA splicing and in the regulation of alternative |
|  |  |  |  |  | splicing events. Activates exon skipping of its own |
|  |  |  |  |  | pre-mRNA during muscle cell differentiation. Binds to |
|  |  |  |  |  | the polypyrimidine tract of introns. May promote RNA |
|  |  |  |  |  | looping when bound to two separate polypyrimidine |
|  |  |  |  |  | tracts in the same pre-mRNA. May promote the binding |
|  |  |  |  |  | of U2 snRNP to pre-mRNA. Cooperates with RAVER1 |
|  |  |  |  |  | to modulate switching between mutually exclusive |
|  |  |  |  |  | exons during maturation of the TPM1 pre-mRNA. |
|  |  |  |  |  | Represses the splicing of MAPT/Tau exon 10 |
| SND1 | 22 | 2.56 | 16 | 0 | staphylococcal nuclease and tudor domain containing 1; |
|  |  |  |  |  | Functions as a bridging factor between STAT6 and the |
|  |  |  |  |  | basal transcription factor. Plays a role in PIM1 |
|  |  |  |  |  | regulation of MYB activity. Functions as a |
|  |  |  |  |  | transcriptional coactivator for the Epstein-Barr virus |
|  |  |  |  |  | nuclear antigen 2 (EBNA2) |
|  |  |  |  |  |  |

**Supplemental Figures**


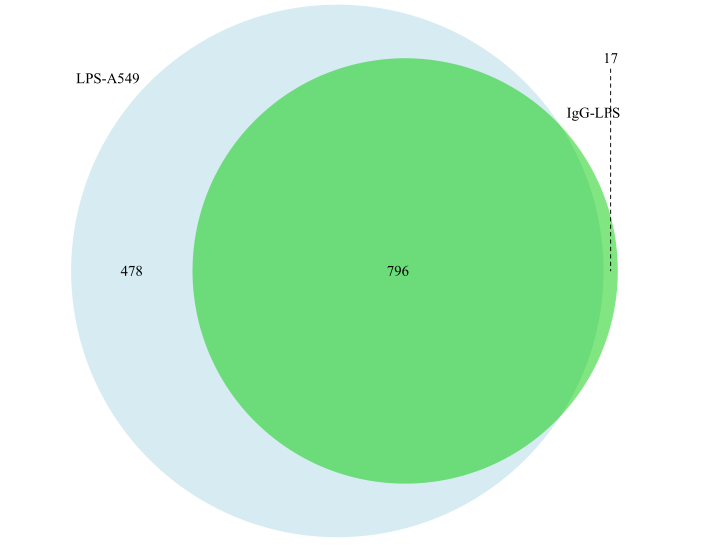


Figure S1. Venn diagram of the different proteins in LPS-A549 vs. IgG-LPS. Venn diagram of the different proteins in LPS-A549 vs. IgG-LPS. The green part represents proteins enriched in IgG-LPS; The light blue represents proteins enriched in LPS-A549; The middle part is the protein identified by both of them.


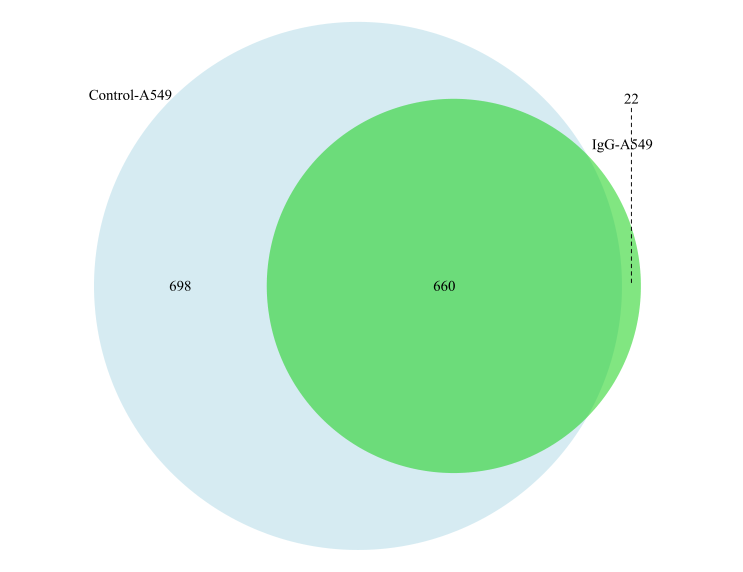


Figure S2. Venn diagram of the different proteins in control-A549 vs. IgG-A549. The green part represents proteins enriched in IgG-A549; The light blue represents proteins enriched in Control-A549; The middle part is the protein identified by both of them.


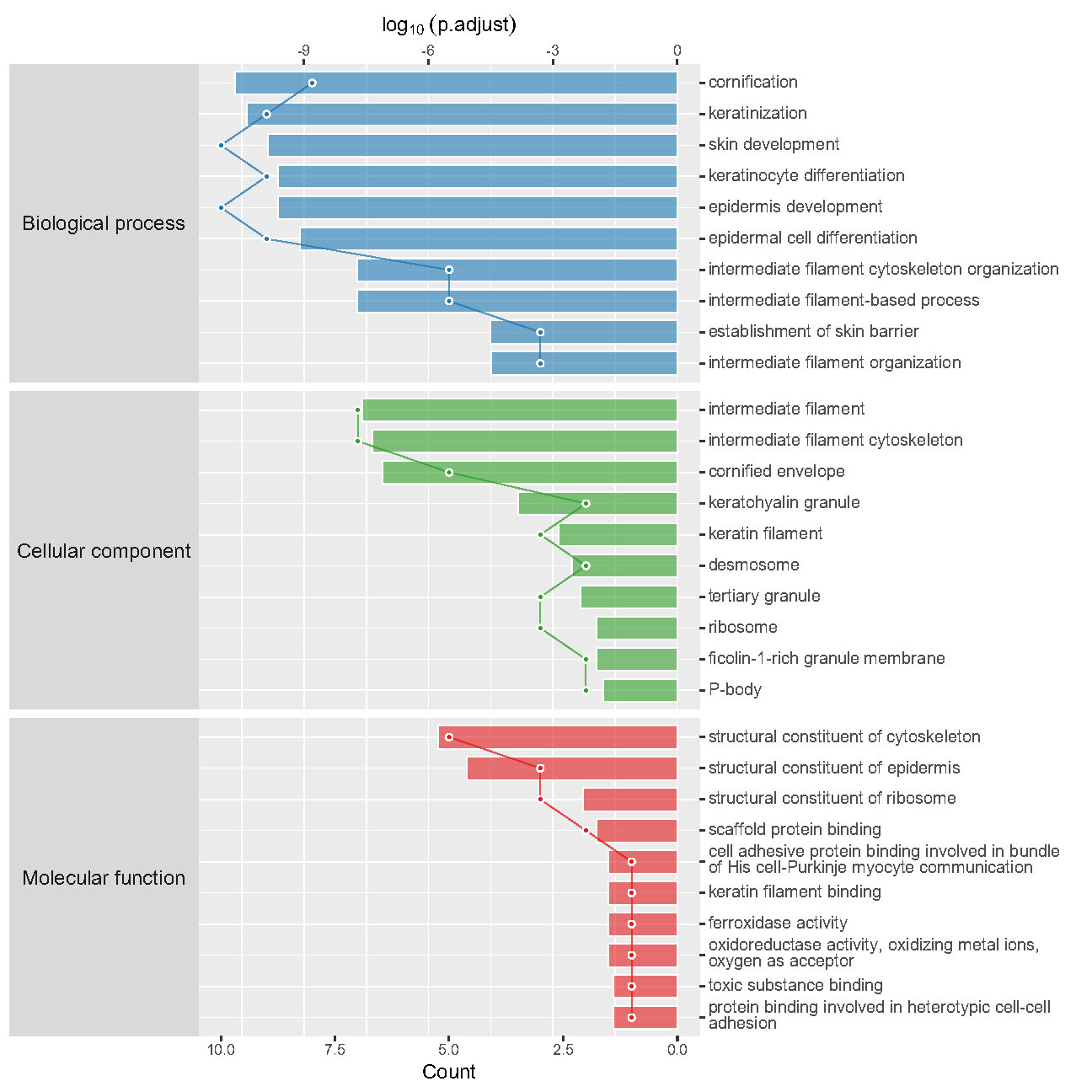


Figure S3. Enriched GO items of < C > in Control-A549 vs. IgG-A549. top axis is log10(adjust p-value), bottom axis is gene count.


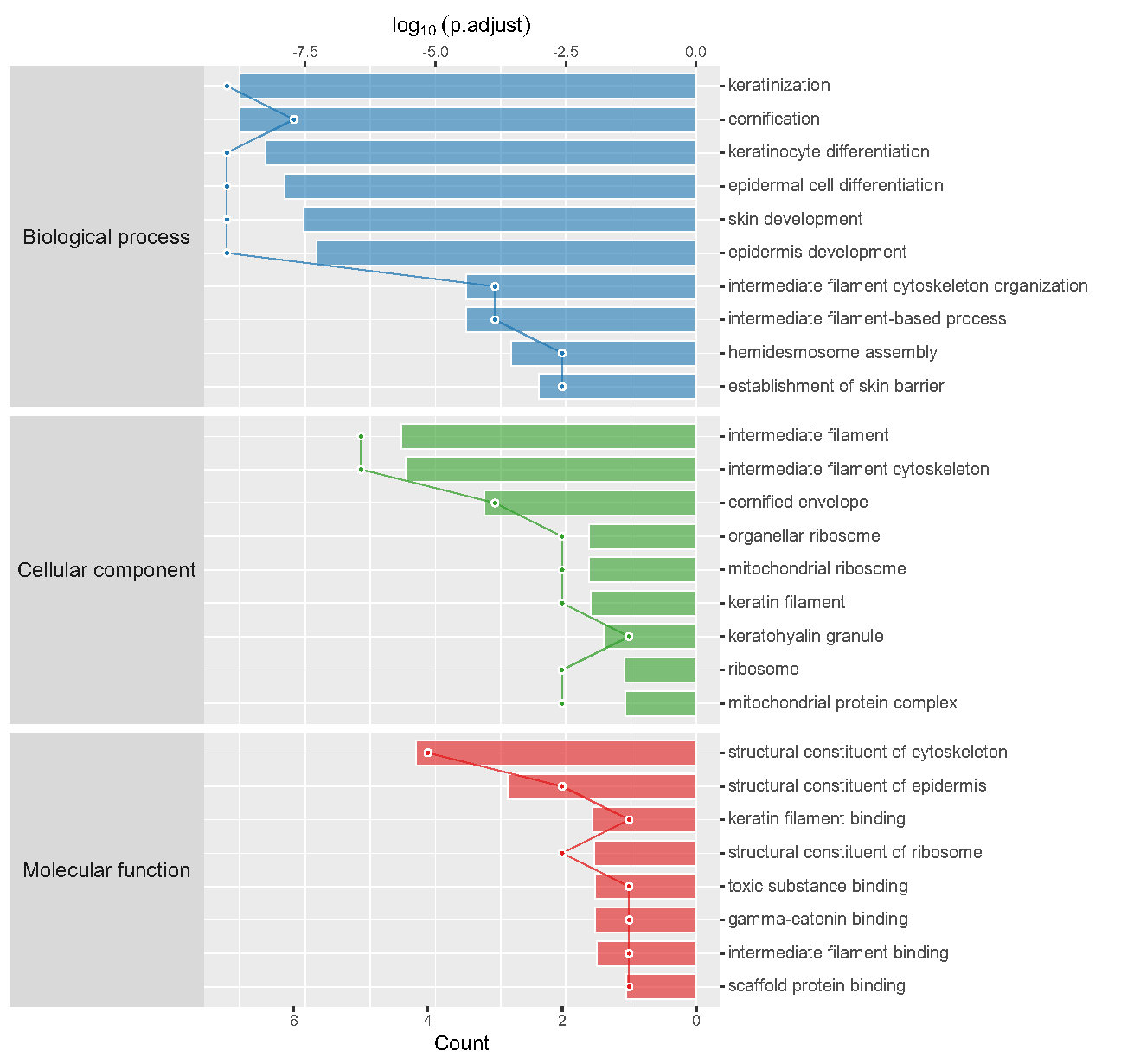


Figure S4. Enriched GO items of < C > in LPS-A549 vs. IgG-LPS. top axis is log10(adjust p-value), bottom axis is gene count.


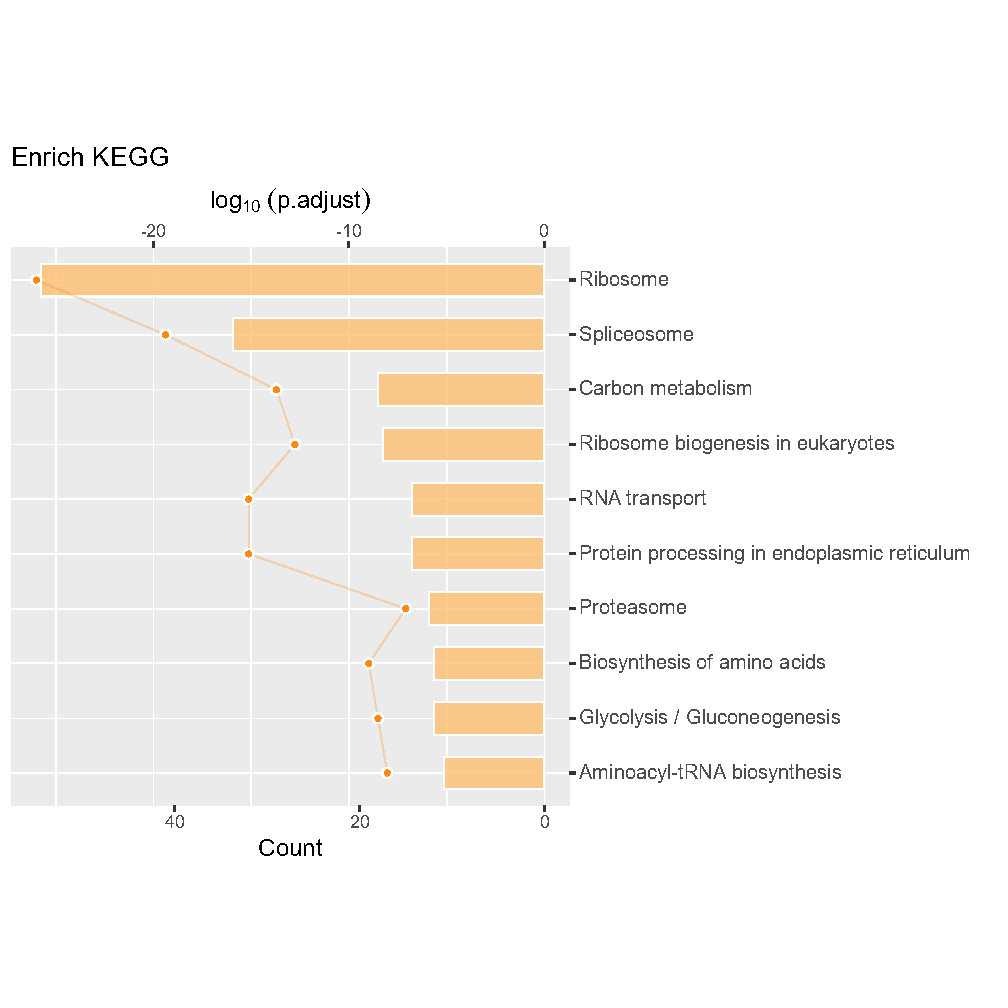


Figure S5. Enriched KEGG items of < T > in Control-A549 vs. IgG-A549, if no protein matched or less than 5 enriched items found, on plot will be displayed.


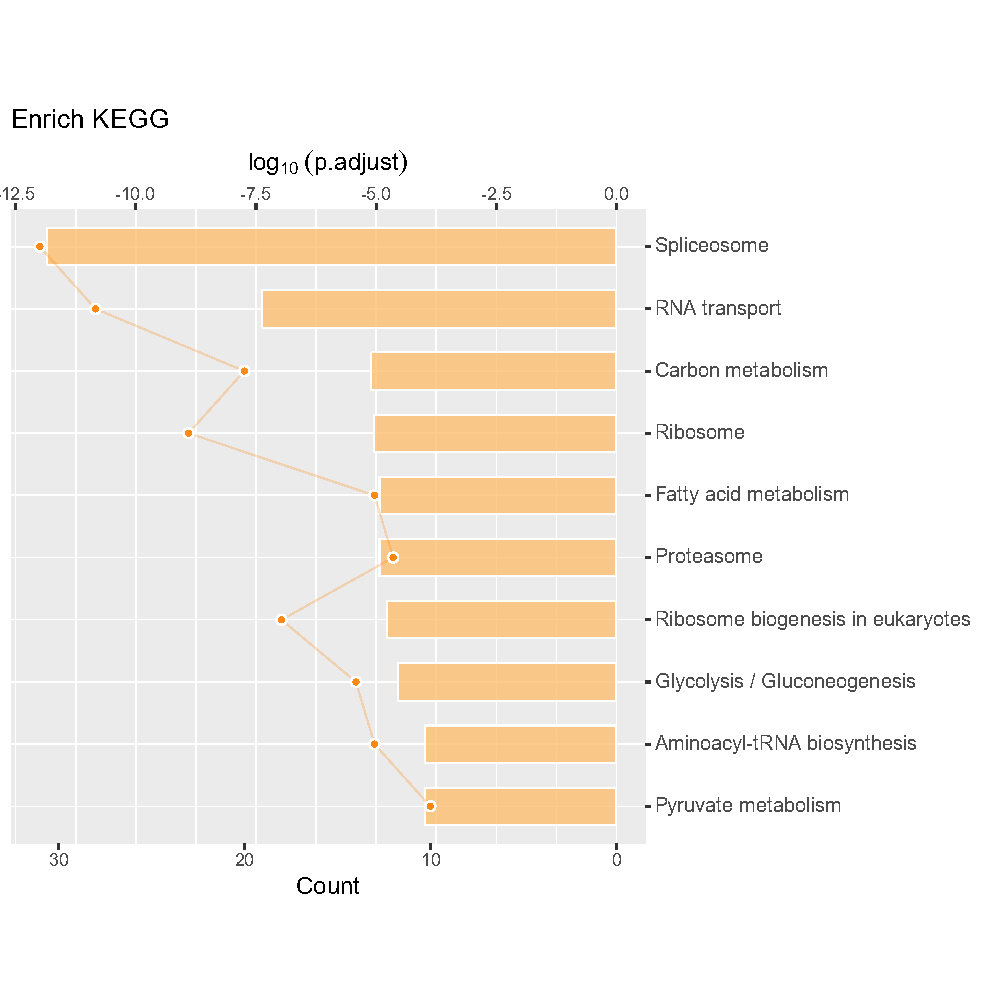


Figure S6. Enriched KEGG items of < T > in LPS-A549 vs. IgG-LPS, if no protein matched or less than 5 enriched items found, on plot will be displayed.


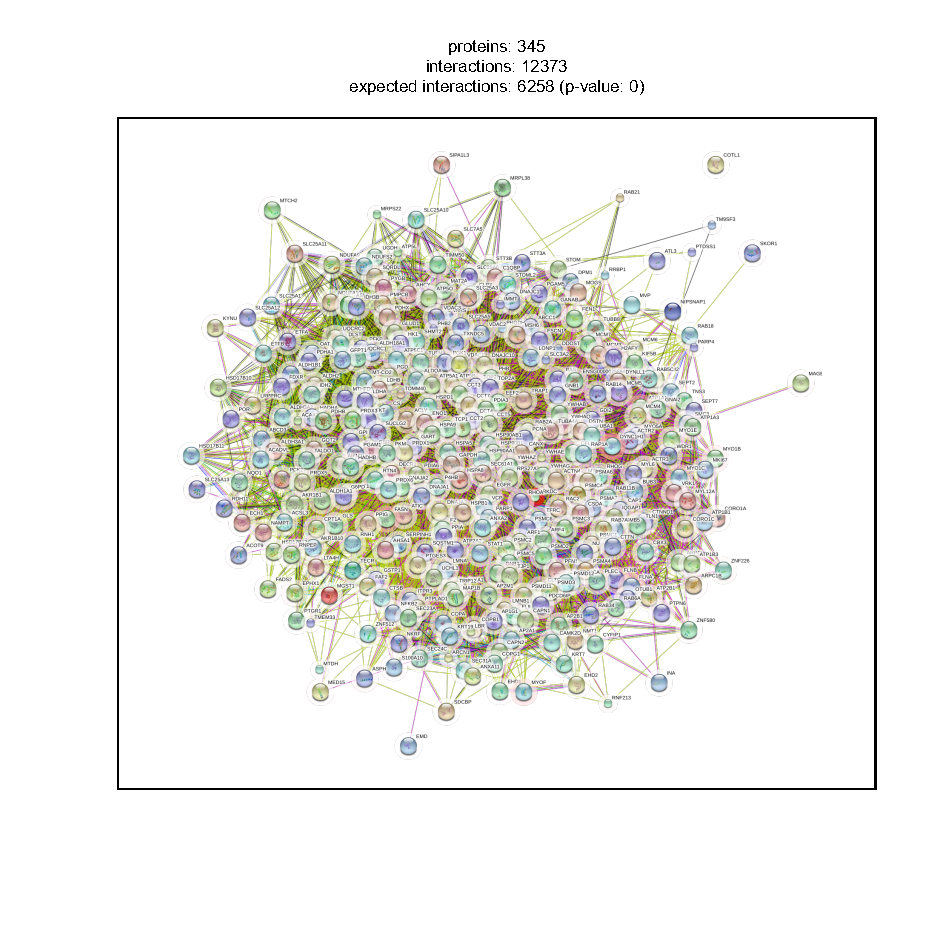
Figure S7. Control-A549--IgG-A549-STRINGdb-T-1.


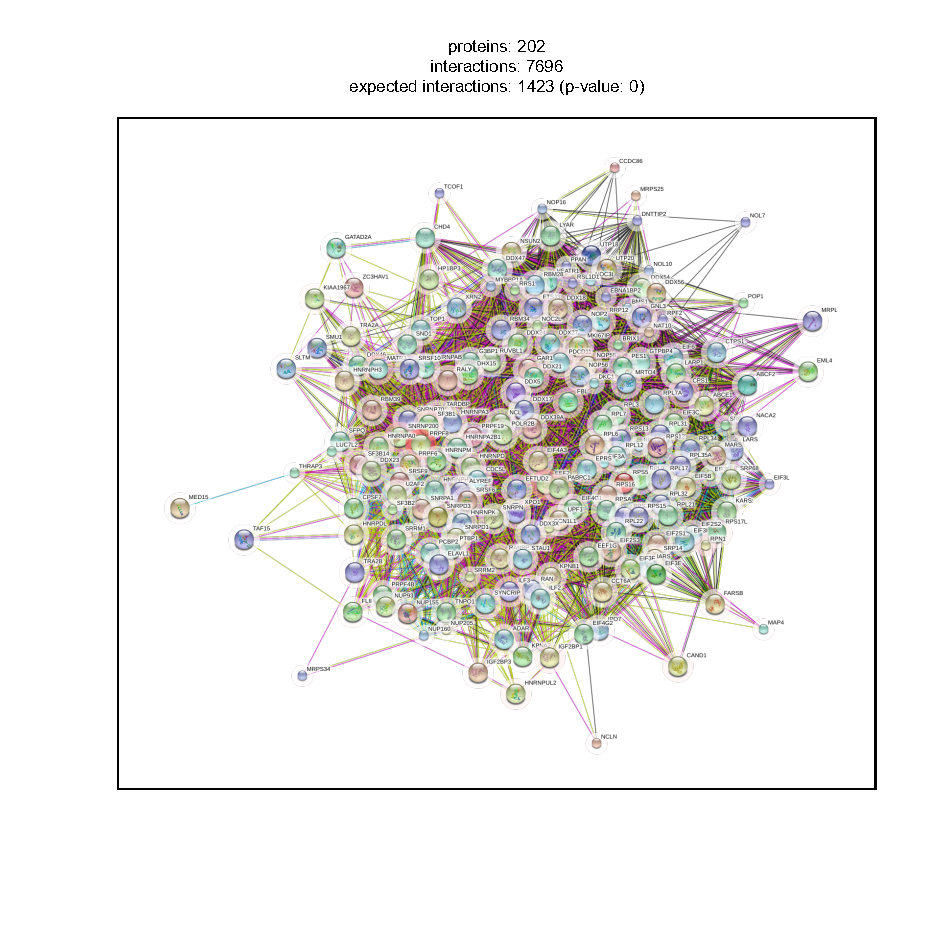
Figure S8. LPS-A549--IgG-LPS-STRINGdb-T-1
